# Learning from prepandemic data to forecast viral escape

**DOI:** 10.1038/s41586-023-06617-0

**Published:** 2023-10-11

**Authors:** Nicole N. Thadani, Sarah Gurev, Pascal Notin, Noor Youssef, Nathan J. Rollins, Daniel Ritter, Chris Sander, Yarin Gal, Debora S. Marks

**Affiliations:** 1 Marks Group, Department of Systems Biology, Harvard Medical School, Boston, MA USA; 2 Department of Electrical Engineering and Computer Science, MIT, Cambridge, MA USA; 3 OATML Group, Department of Computer Science, University of Oxford, Oxford, UK; 4 Broad Institute of Harvard and MIT, Cambridge, MA USA; 5 Present Address: Seismic Therapeutic, Watertown, MA USA

**Keywords:** Immune evasion, Computational models

## Abstract

Effective pandemic preparedness relies on anticipating viral mutations that are able to evade host immune responses to facilitate vaccine and therapeutic design. However, current strategies for viral evolution prediction are not available early in a pandemic—experimental approaches require host polyclonal antibodies to test against^1–16^, and existing computational methods draw heavily from current strain prevalence to make reliable predictions of variants of concern^17–19^. To address this, we developed EVEscape, a generalizable modular framework that combines fitness predictions from a deep learning model of historical sequences with biophysical and structural information. EVEscape quantifies the viral escape potential of mutations at scale and has the advantage of being applicable before surveillance sequencing, experimental scans or three-dimensional structures of antibody complexes are available. We demonstrate that EVEscape, trained on sequences available before 2020, is as accurate as high-throughput experimental scans at anticipating pandemic variation for SARS-CoV-2 and is generalizable to other viruses including influenza, HIV and understudied viruses with pandemic potential such as Lassa and Nipah. We provide continually revised escape scores for all current strains of SARS-CoV-2 and predict probable further mutations to forecast emerging strains as a tool for continuing vaccine development (evescape.org).

## Abstract (web summary)

EVEscape, a flexible framework using deep learning and biophysical structural information, enables early identification of concerning mutations in viruses with pandemic potential, facilitating the development of vaccines and therapeutics.

## Main

Viral diseases involve a complex interplay between immune detection in the host and viral evasion, often leading to the evolution of viral antigenic proteins. Antibody escape mutations affect viral reinfection rates and the duration of vaccine efficacy. Therefore, anticipating viral variants that avoid immune detection with sufficient lead time is key to developing optimal vaccines and therapeutics.

Ideally, we would be able to anticipate viral immune evasion using experimental methods such as pseudovirus assays^1^ and higher-throughput deep mutational scans^2–16^ (DMSs) that measure the ability of viral variants to bind to relevant antibodies. However, these experimental methods require antibodies or sera representative of the aggregate immune selection imposed on the virus, which become available only as large swaths of the population are infected or vaccinated, limiting the impact for early prediction of immune escape. In addition, as pandemic viruses can evolve rapidly (tens of thousands of new SARS-CoV-2 variants are sequenced each month), systematically testing all variants as they emerge is intractable, even without considering the effects of potential mutations on circulating strains.

It is therefore of interest to develop computational methods for predicting viral escape that can be used to identify mutations that may emerge. An ideal model would be able to assess escape likelihood for as-yet-unseen variation throughout the full antigenic protein, would inform the design of targeted experiments, would be revised with pandemic information and would make predictions with sufficient lead time for vaccine development (that is, before immune responses to the virus are observed). However, previous computational methods for forecasting viral fitness or immune escape depend critically on real-time sequencing or pandemic antibody structures, limiting their ability to predict unseen variants and making them impractical for vaccine development during the onset of a pandemic^17–19^.

In this work, we introduce EVEscape, a flexible framework that addresses the weaknesses of previous methods by combining a deep generative model trained on historical viral sequences with structural and biophysical constraints. Unlike previous methods, EVEscape does not rely on recent pandemic sequencing or antibodies, making it applicable both in the early stages of a viral outbreak and for continuing evaluation of emerging SARS-CoV-2 strains. By leveraging functional constraints learned from past evolution, as successfully demonstrated for predicting clinical variant effects^20–22^, EVEscape can capture relevant epistasis^23–25^ and thus predict mutant fitness in the context of any strain background. Moreover, EVEscape is adaptable to new viruses, as we demonstrate in both our validation on SARS-CoV-2, HIV and influenza and in predictions for the understudied Nipah and Lassa viruses. This approach enables advance warning of concerning mutations, facilitating the development of more effective vaccines and therapeutics. Such an early warning system could guide public health decision-making and preparedness efforts, ultimately minimizing the human and economic impact of a pandemic.

## EVEscape combines deep learning models and biophysical constraints

**Fig. 1:**
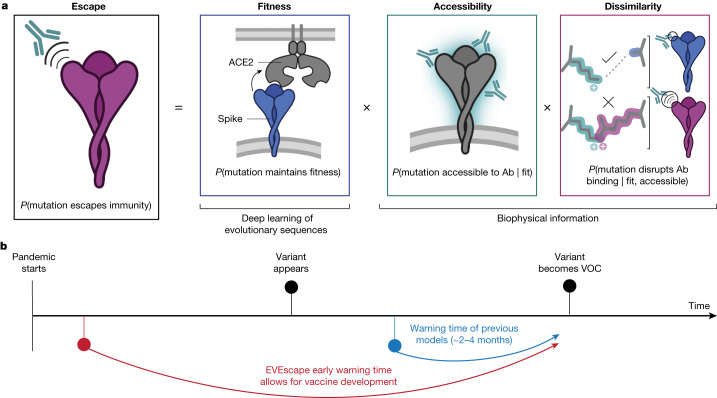
Early prediction of antibody escape from deep generative sequence models, structural and biophysical constraints. **a**, EVEscape assesses the likelihood of a mutation escaping the immune response on the basis of the probabilities of a given mutation maintaining viral fitness, occurring in an antibody epitope and disrupting antibody binding. **b**, EVEscape requires only information available early in a pandemic, before surveillance sequencing, antibody–antigen structures or experimental mutational scans are broadly available. This provides further early warning time critical for vaccine development. Ab, antibody. Panel **a** created with BioRender.com.

**Extended Data Fig. 1:**
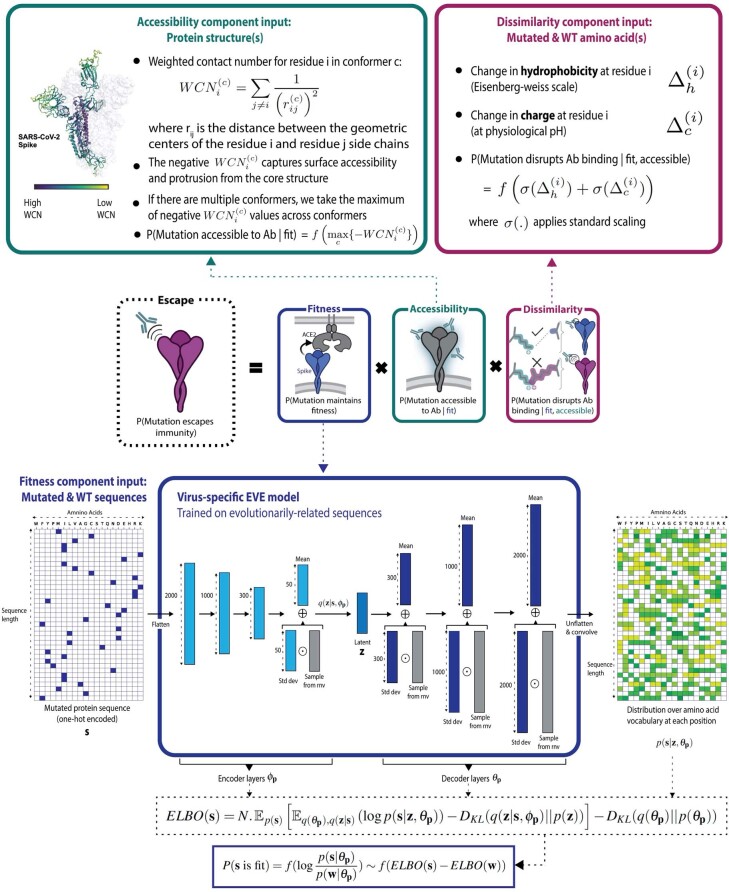
EVEscape model components. We decompose the likelihood of a mutation to escape the immune response as the product of three components: probability of a given mutation to maintain viral fitness (fitness component), to occur in an antibody epitope (accessibility component), and to disrupt antibody binding (dissimilarity component). For fitness (bottom), we train a virus-specific Bayesian VAE on evolutionarily-related proteins to learn a distribution over sequences in that protein family. The ELBO term from the VAE is used as a tractable approximation to the sequence log likelihood, with Δ ELBOs thus quantifying the relative fitness of a given mutated sequence **s** with respect to the wild type **w**. Accessibility (top left) is quantified via the negative Weighted Contact Number (WCN) for a residue in a given conformation. If there are multiple conformations, the maximum negative WCN across conformations is used. Dissimilarity (top right) relies on change in key physicochemical properties induced by the mutation, such as hydrophobicity and charge. For all components, the operator *f*(.) represents a component-specific temperature-scaled logistic transform. Created with BioRender.com.

Viral proteins that escape humoral immunity disrupt polyclonal antibody binding while retaining protein expression, protein folding, host receptor binding and other properties necessary for viral infection and transmission^8^. We built a modelling framework, EVEscape, that incorporates constraints from these different aspects of viral protein function learned from different data sources. We express the probability that a mutation will induce immune escape as the product of three probabilities: the likelihoods that a mutation will maintain viral fitness (‘fitness’ term), occur in an antibody-accessible region (‘accessibility’ term) and disrupt antibody binding (‘dissimilarity’ term) (Fig. 1a and Extended Data Fig. 1). These components are amenable to prepandemic data sources, allowing for early warning (Fig. 1b).

**Extended Data Fig. 2:**
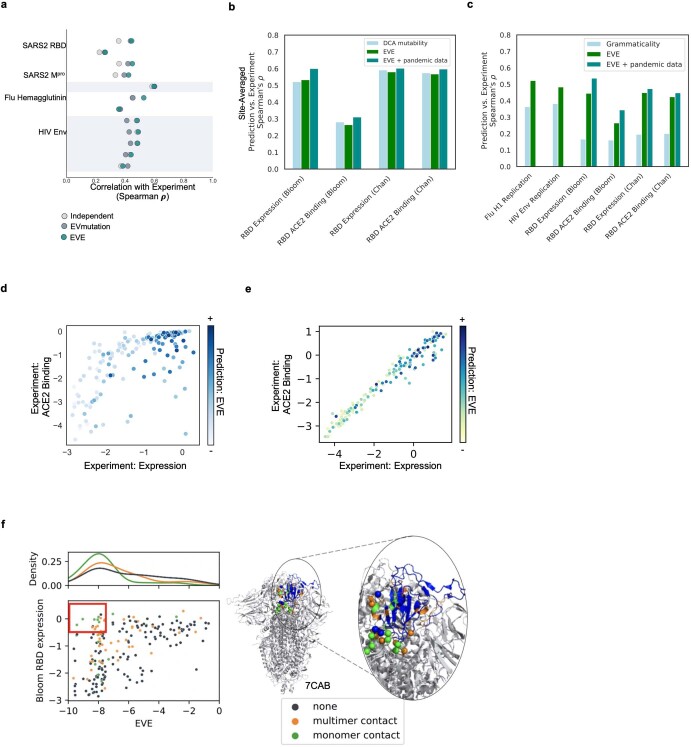
Fitness effects of viral proteins predicted from evolutionary sequence models. a) EVE predictions are well correlated with a broad range of viral surface protein deep mutation scanning experiments surveying protein replication and function for SARS-CoV-2 RBD^30,31^ and M^pro32^, H1N1 hemagglutinin^26,27^ and HIV env^25,28,29^. b) Site-averaged EVE predictions have similar correlations with site-averaged SARS-CoV-2 RBD DMS experiments as Potts model DCA^37^ or EVmutation^21^. c) EVE predictions have higher correlations with Flu H1, HIV Env, and SARS-CoV-2 RBD DMS experiments than grammaticality in CSCS^42^. d) EVE prediction captures a combination of SARS-CoV-2 RBD yeast expression and ACE2 binding - features both necessary for successful immune escape (EVE spearman with expression = 0.45, EVE spearman with ACE2 binding = 0.38 when low expression mutations are removed)^30^. e) The mammalian-cell RBD expression and ACE2 binding experiments are highly correlated, likely due to the alternate FACS-binning strategy and metric used for this ACE2 binding experiment^31^. EVE predictions are correlated with both measures. f) Site-averaged EVE scores predict several sites that tolerate mutants in the yeast-display RBD expression assay^30^ to be deleterious (red box)–many of these mutants are located at the interface between RBD and the rest of Spike protein. Sites in the red box in scatterplot are shown as spheres on the Spike structure (PDB: 7CAB).

First, we estimated the fitness effect of substitution mutations (subsequently referred to as mutations) using EVE^20^, a deep variational autoencoder trained on evolutionarily related protein sequences (Supplementary Tables 1 and 2) that learns constraints underpinning structure and function for a given protein family. Consequently, EVE considers dependencies across positions (epistasis), capturing the changing effects of mutations as the dominant strain backgrounds diversify from the initial sequence^23–25^. We demonstrate the efficacy of EVE by comparing model predictions and data from mutational scanning experiments that measure several facets of fitness for thousands of mutations to viral proteins^25–32^. Model performance approaches the Spearman correlation (*ρ*) between experimental replicates, including viral replication for influenza^26^ (*ρ* = 0.53) and HIV^25^ (*ρ* = 0.48) (Extended Data Fig. 2 and Supplementary Tables 3 and 4). For SARS-CoV-2, we trained EVE across broad prepandemic coronavirus sequences, from sarbecoviruses including SARS-CoV-1 to ‘common cold’ seasonal coronaviruses including the alphacoronavirus NL63 (Supplementary Tables 1 and 2), and compared predictions with measures of expression (*ρ* = 0.45) and receptor binding^30^ (*ρ* = 0.26) (Extended Data Fig. 2 and Supplementary Table 4). We note that sites that expressed in the DMS experiments but were predicted to be deleterious by EVE were frequently in contact with non-assayed domains of the Spike protein or with the trimer interface, interactions not captured in the receptor-binding domain (RBD) yeast-display experiment (Extended Data Fig. 2f).

**Extended Data Fig. 3:**
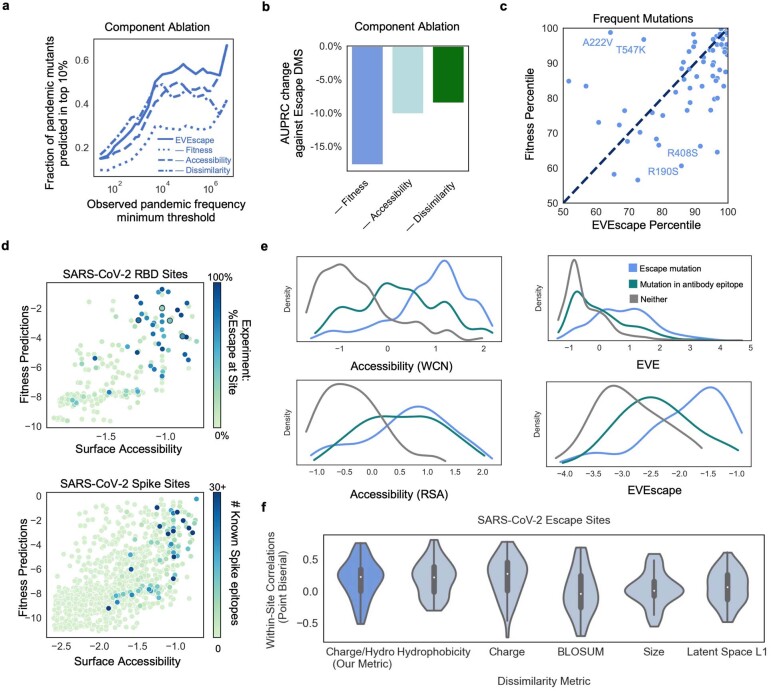
Understanding the roles of each EVEscape component. a) EVEscape is more predictive of high-frequency pandemic mutations than ablations of any of its three components. Notably, the ablation of the dissimilarity term leads to similar performance at identifying low-frequency mutations, but inferior performance at identifying high-frequency mutations. b) Ablation analysis indicates that all features of EVEscape contribute to performance in predicting RBD escape mutants in deep mutational scanning experiments. c) EVEscape is more predictive than EVE alone at capturing frequent mutations (seen >50,000 times) in full Spike. VOC mutations with high EVE scores and lower EVEscape scores (i.e., A222V and T547K) are known to impact protein conformation and to not escape sera neutralization^39^. Mutations with the highest EVEscape but low EVE scores (i.e., R190S and R408S) are in hydrophobic pockets that may promote antibody binding^38^. d) Sites with either high WCN accessibility or high EVE fitness predictions have a greater percent of escape mutants (upper). WCN and EVE predictions provide similar information about the location of Spike epitopes as identified from antibody-Spike crystal structures in RCSB PDB (lower). e) Density of standard-scaled EVEscape components differ for SARS-CoV-2 RBD escape (and antibody epitope) mutations and non-escape mutations for WCN, RSA, EVE, and site-averaged EVEscape. All but 2 sites in the top 20% of EVEscape scores are in known antibody footprints or have escape mutations in experiments. f) Within-site point biserial correlations between residue dissimilarity metrics and SARS-CoV-2 DMS escape data at escape sites (sites with 3–17 escape mutations). More sites have a higher correlation for our charge-hydrophobicity metric than charge or hydrophobicity alone, BLOSUM62, residue size, or EVE latent space L1 distance. Bounds of boxplot are quartiles with the median as the measure of center.

The second model component, antibody accessibility, is motivated by the need to identify potential antibody binding sites without previous knowledge of B cell epitopes. The accessibility of each residue is computed from its negative weighted residue-contact number across available three-dimensional conformations (without antibodies), which captures both protrusion from the core structure and conformational flexibility^33^ (Supplementary Table 1). Finally, dissimilarity is computed using differences in hydrophobicity and charge, properties known to affect protein–protein interactions^34^. This simple metric correlates with experimentally measured within-site escape more than individual chemical properties, substitution-matrix derived distance or distance in the latent space of the EVE model (Extended Data Fig. 3f). To support modularity and interpretability of the impact of each component, each term is separately standardized and then fed into a temperature-scaled logistic function (Supplementary Methods and Supplementary Tables 5 and 6).

## Anticipating pandemic variation with prepandemic data

Extensive surveillance sequencing and experimentation prompted by the COVID-19 pandemic have presented a unique opportunity to assess the ability of EVEscape to predict immune evasion before escape mutations are observed. To test the model’s capacity to make early predictions, we carried out a retrospective study using only information available before the pandemic (training on Spike sequences across *Coronaviridae* available before January 2020; Supplementary Tables 1 and 2). We then evaluated the method by comparing predictions against what was subsequently learned about SARS-CoV-2 Spike immune interactions and immune escape.

**Fig. 2:**
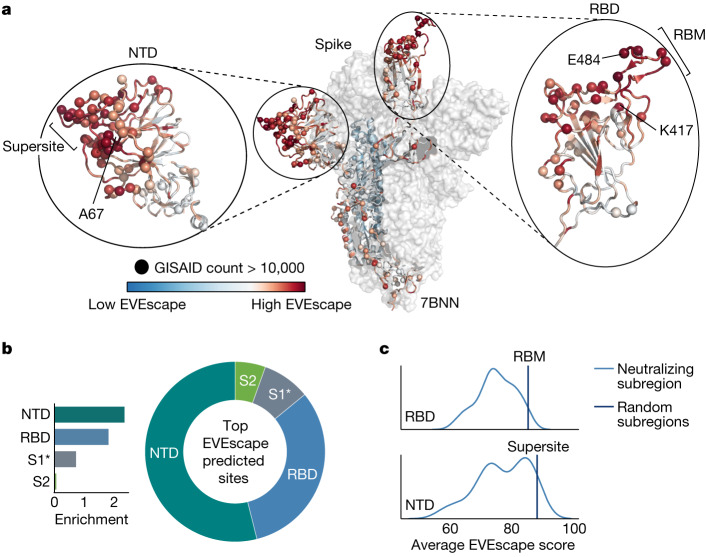
EVEscape identifies antigenic regions without antibody information. **a**, EVEscape scores (site-level maximum) mapped onto a representative Spike three-dimensional structure (Protein Data Bank (PDB) identifier: 7BNN) highlight high-scoring regions with many observed pandemic variants, both in the RBD and in the NTD. Spheres indicate sites with a total number of mutations observed more than 10,000 times in the GISAID sequence database. **b**, The top decile of EVEscape predictions span diverse epitope regions across Spike, but most of the predictions are in the NTD and RBD, which have a disproportionately high number of predicted EVEscape sites relative to their sequence length (enrichment). The regions considered are NTD (sequence positions 14–306), RBD (319–542), S1* (543–685) and S2 (686–1273), where S1* refers to the region in S1 between RBD and the S2. **c**, Neutralizing subregions—RBM (receptor-binding motif, 438–506) and NTD supersite^36^ (14–20,140–158, 245–263)—have significantly higher than average EVEscape scores, relative to a distribution of 150 random contiguous regions of the same length within the RBD and NTD, respectively. Source data

**Extended Data Fig. 4:**
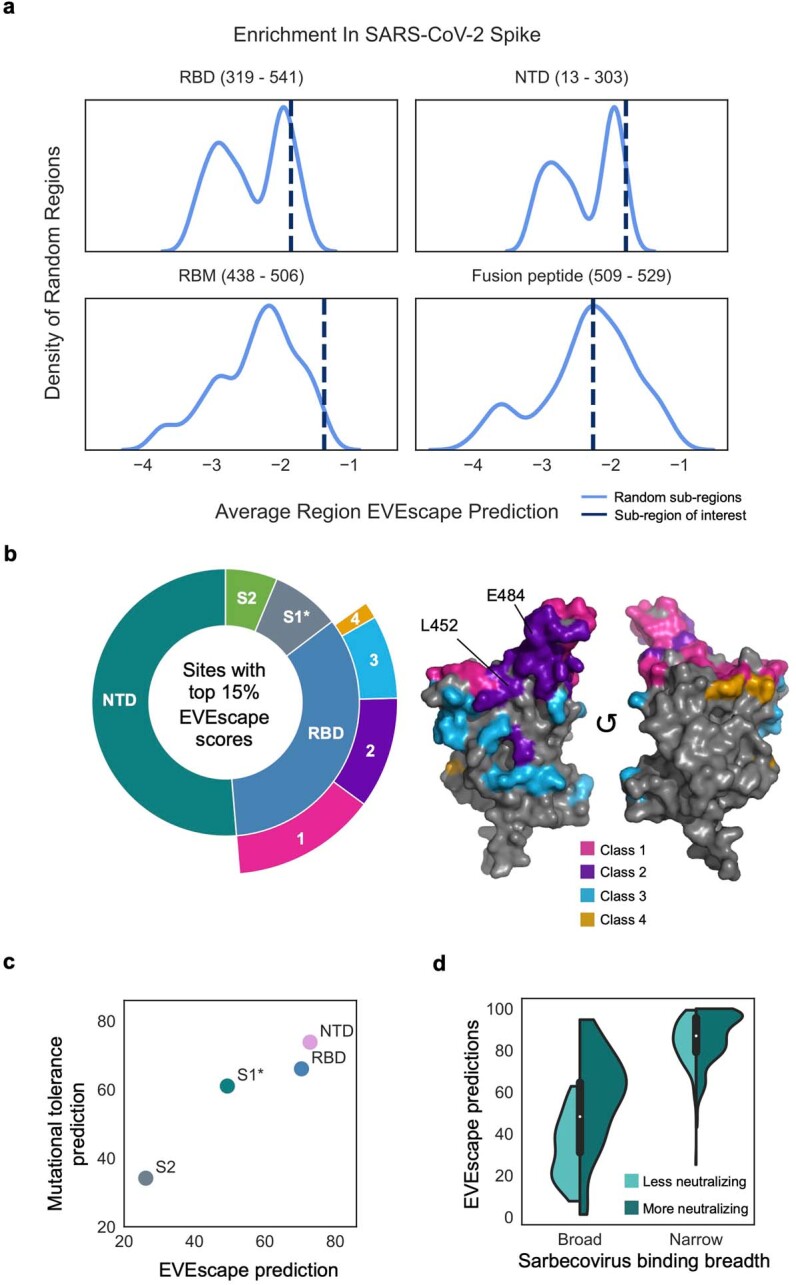
EVEscape enrichment in regions of SARS-CoV-2 Spike. a) RBD (particularly receptor binding motif (RBM)) and N-terminal domain (NTD) have significantly enriched average EVEscape scores, relative to a distribution of 500 random contiguous regions of the same length from full Spike. Fusion peptide (not known for escape mutations) does not have enriched average EVEscape scores. b) EVEscape predictions cover diverse epitope regions across Spike and diverse RBD antibody classes (Supplementary Methods) (3D structure of RBD on the right), including known immunodominant sites (E484, K417, L452) (PDB ID: 7BNN). The regions considered are NTD (sequence positions 14 − 306), RBD (319 − 542), S1* (543 − 685), and S2 (686 − 1273), where S1* refers to the region in S1 between RBD and S2. c) Average region EVEscape predictions are highest in RBD and NTD, although NTD is more mutationally tolerant based on average fitness (EVE) score. d) EVEscape scores experimental escape mutants from narrow antibodies and broad neutralizing antibodies higher than those from broad, non-neutralizing antibodies. Sarbecovirus binding breath and neutralization from Starr et al. ^8^ Bounds of boxplot are quartiles with the median as the measure of center.

The top predicted escape mutations for the whole of Spike were strongly biased towards the RBD and N-terminal domain (NTD), coincident with the bias for antigenic regions seen in the pandemic^35^ (Fig. 2a,b and Extended Data Fig. 4). Within these domains, EVEscape scores were biased towards neutralizing regions—the receptor-binding motif of the RBD and the neutralizing supersite^36^ in the NTD (Fig. 2c and Extended Data Fig. 4d). The ability of EVEscape to identify the most immunogenic domains of viral proteins without knowledge of specific antibodies or their epitopes could provide crucial information for early development of subunit vaccines in an emerging pandemic.

We next compared model predictions with mutations that were subsequently observed in the pandemic as deposited in GISAID (Global Initiative on Sharing All Influenza Data), which contains more than 750,000 unique sequences. For this analysis, we focused on the RBD of Spike, as this domain has been the most extensively studied owing to its immunodominance^35^.

**Fig. 3:**
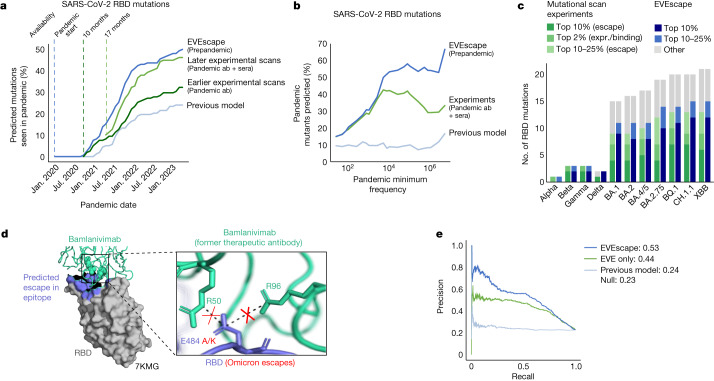
Prepandemic EVEscape is as accurate as intrapandemic experimental scans at anticipating pandemic variation. **a**, Percentages of top decile predicted escape mutations by EVEscape, mutational scan experiments (Bloom Set, Supplementary Table 5) and a previous computational model^42^ seen more than 100 times in GISAID by each date since the start of the pandemic. EVEscape using prepandemic sequences anticipates pandemic variation at least on par with mutational scan experiments using antibodies and sera available 10 or 17 months into the pandemic. Analysis focuses only on non-synonymous point mutations that are a single nucleotide distance away from the Wuhan viral sequence, as well as on the RBD of Spike as that is where experimental data are available. **b**, Percentages of observed pandemic mutations in top decile of escape predictions by observed frequency during the pandemic. High-frequency mutations in particular are well-captured by EVEscape. **c**, Most of the RBD mutations observed in VOC strains have high EVEscape scores and lower scores in the mutational scan experiments against pandemic sera. This is true even when considering a further set of mutations identified in mutational scanning experiments as significantly improving (in the top 2%) either RBD expression or ACE2 binding. **d**, EVEscape can predict escape mutations in the epitope of the former therapeutic antibody bamlanivimab. E484 is involved in a salt bridge with R96 and R50 of bamlanivimab, which lost Food and Drug Administration emergency use authorization owing to the emergence of Omicron, wherein E484A or E484K mutations (both predicted in the top 1% of EVEscape Spike predictions) escape binding because of the loss of these salt bridges^41^.**e**, Precision-recall curve for RBD escape predictions of EVEscape, EVEscape fitness component only (EVE model) and a previous computational model^42^ compared with DMS escape mutations (AUPRC reported with a comparison with a ‘null’ model in which escape mutations are randomly predicted). expr, expression.; no., number. Source data

**Extended Data Fig. 5:**
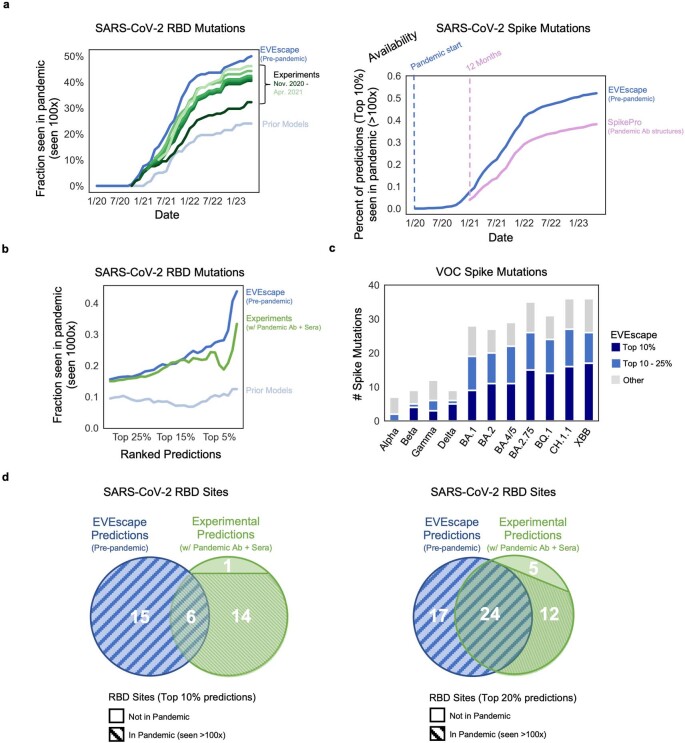
EVEscape as accurate as experimental scans at anticipating pandemic variation: retrospective analysis. a) Top 10% of RBD escape predictions computed using either EVEscape, DMS experiments (Bloom Set, Table S4), or prior models^42^ seen by each date over 100 times in GISAID (left). DMS experiments are separated into which studies were available by each starting date. Top 10% of full Spike escape predictions computed using either EVEscape or prior SpikePro model^18^ seen by each date over 100 times in GISAID (right). b) Fraction of top mutations (at different percentage thresholds) predicted by EVEscape, DMS experiments, or prior models seen more than 1000 times in GISAID. c) The majority of Spike mutations in VOC strains have high EVEscape scores. d) Venn diagram comparing the top 10% (left) or 20% (right) of RBD sites predicted by EVEscape and by DMS experiments (Bloom Set Table S4). Each bin is stratified to indicate the number of sites observed >100 times over the full pandemic (stripe pattern). All sites in the top 20% of EVEscape predictions have been observed in the pandemic, and there is significantly more overlap between EVEscape and experiments when looking at the top 20% of their predictions as compared to the top 10%.

Fifty percent of our top RBD predictions were seen in the pandemic by May 2023 (Fig. 3a; this proportion is robust to the threshold defining top escape mutations). The more often a mutation occurred in the pandemic, the more likely it was to be predicted by our method—66% of high-frequency observed substitutions were in the top EVEscape predictions (Fig. 3b). We expect that the highest-frequency mutations, seen in historical variants of concern (VOCs), will be enriched for escape variants that provide a fitness advantage in an immune population (while not expecting that all single substitutions in the VOCs will contribute to escape) (Fig. 3c and Extended Data Fig. 5).

Not surprisingly, the fitness model component alone (here EVE^20^) was better that the full EVEscape model at predicting mutations seen at low frequency in the pandemic (that is, identifying 357 versus 298 of mutations seen 100–1,000 times in the pandemic in the top quartile), probably because these mutations retain viral function but do not necessarily affect antibody binding or have a strong fitness advantage over other strains. This indicates that the immune-specific components of EVEscape may reflect important pandemic constraints not represented in models of fitness alone^20,37^ and allow for mutation interpretability. For instance, VOC mutations R190S and R408S, with high EVEscape but low EVE scores, are in hydrophobic pockets that may facilitate significant immune escape^38^ (Extended Data Fig. 3c). Meanwhile, the few VOC mutations (A222V and T547K) with significant EVE—but not EVEscape—scores have known functional improvements such as monomer packing and RBD opening but do not affect escape^39,40^ (Extended Data Fig. 3c). Furthermore, the proportion of EVEscape predictions seen during the pandemic increased over time—from 3% in December 2020 to 50% in May 2023 (Fig. 3a)—and should continue to increase, an expected trend both as more variants are observed and as adaptive immune pressure increases with the growing vaccinated or previously infected population. Similarly, the fraction of mutations in VOC strains with high EVEscape scores has also increased over time (Fig. 3b).

Our model also predicted escape mutations that were subsequently observed in the pandemic in the epitopes of well-known therapeutic monoclonal antibodies under current or former emergency use authorization (Supplementary Table 7), for example, N440, E484A/K/Q and Q493R. These predictions demonstrate the interplay of our three model components; for instance, the high accessibility as well as mutability of E484 results in 50% of all possible mutations at this site in the top 2% of EVEscape predictions and includes E484A/K mutations in the top 1%—notable for escape from bamlanivimab^41^ (Fig. 3d)—because of their high dissimilarity scores. We also identify candidate escape mutations in these therapeutic epitopes that have not yet been observed at frequencies higher than 10,000—for instance variants to K444 and K417 (Supplementary Table 7), a subset of which are beginning to appear. This result indicates that escape sites could be well predicted before a pandemic and may have concrete applications for escape-resistant therapeutic design and early warning of waning effectiveness.

EVEscape represents a significant improvement over past computational methods. EVEscape is more than twice as predictive as previous unsupervised models^42^, both at predicting pandemic mutations (50% versus 24% of top predictions observed in the pandemic and 66% versus 17% of highest-frequency mutations predicted) and at anticipating experimental measures of antibody escape (0.53 versus 0.24 area under the precision-recall curve (AUPRC)) (Fig. 3a–c,e, Extended Data Fig. 5 and Supplementary Tables 4 and 8). All EVEscape components play a part in these predictions, with fitness predictions and accessibility metrics identifying sites of escape mutations, whereas dissimilarity identifies amino acids that facilitate escape within sites (Extended Data Fig. 3). Moreover, other computational methods^18,19^ focus on near-term prediction of strain dominance rather than longer-term anticipation of immune evasion, as they rely on pandemic sequences, antibody-bound Spike structures or both, limiting their early predictive capacity. It is therefore notable that EVEscape outperforms even supervised approaches at predicting mutations seen in the pandemic (Extended Data Fig. 5 and Supplementary Table 8).

## Comparative accuracy of EVEscape and high-throughput experiments

**Extended Data Fig. 6:**
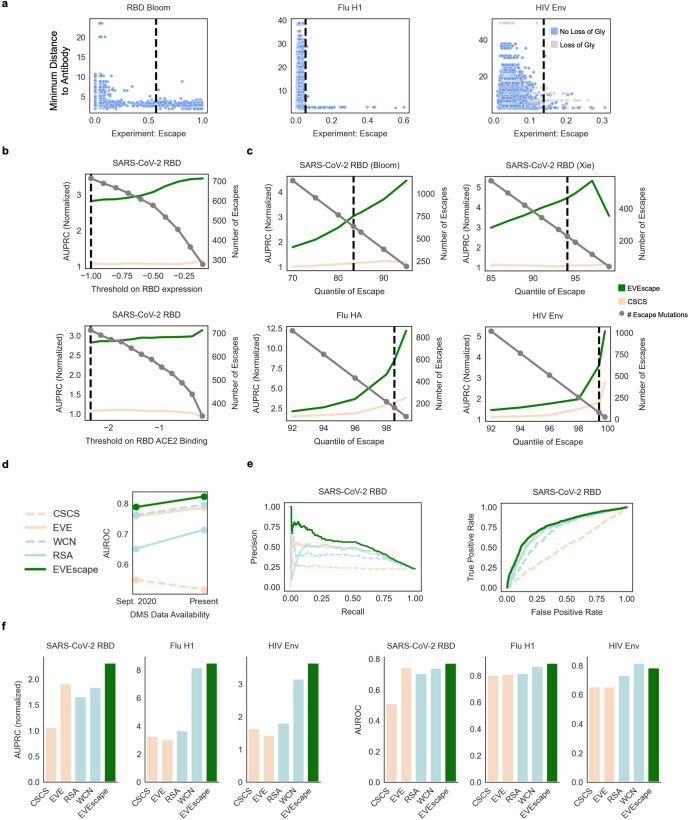
EVEscape comparison to escape deep mutational scans. a) Maximum experimental escape values (over the set of antibodies with PDB structures) for each mutation vs. the minimum distance of the mutation site to a tested antibody—most escape mutations (to the right of dashed line) are to residues with atoms within 5Å of any residue on the antibody. For HIV, this is true for the mutations that do not involve loss of glycosylation. b) Impact of choice of RBD expression and ACE2 binding thresholds (dashed line uses thresholds chosen by Bloom escape papers and our paper) on AUPRC (normalized by “null” model – fraction of observed escapes) and # of mutations considered as escape. c) Impact of choice of escape threshold on RBD (Bloom and Xie data separated), Flu, and HIV AUPRC (normalized) and # of escape mutations (dashed line uses escape threshold chosen by our paper). d) Comparison of model performance (AUROC) between data from first escape DMS study (10 antibodies – Sept. 2020)^3^ and data available at present (338 antibodies, 55 sera samples). e) Precision-Recall curves (normalized by “null” model) (left) and receiver-operator curves (right) for models predicting DMS escape of SARS-CoV-2 RBD. f) AUPRC (normalized by “null” model) (left) and AUROC (right) values for models predicting DMS escape of SARS-CoV-2 RBD, Flu H1, and HIV Env. Note: The “null” model AUPRC is equivalent to the fraction of observed escapes, and therefore AUPRC values are not comparable between viral proteins with different fractions of escape mutations (i.e., SARS-CoV-2 RBD and HIV Env). The fraction of observed escapes in the DMS experiments are 0.19 for RBD, for 0.015 for Flu, and 0.006 for HIV – Flu and HIV data examined far fewer antibody and sera samples (Table S5).

We contextualized the performance of EVEscape in comparison with DMSs, which have been invaluable in identifying and predicting viral variants that may confer immune escape^2–12^. However, these experiments require polyclonal or monoclonal antibodies from infected or vaccinated people, limiting their early predictive capacity. For example, the DMS experiments conducted by 17 months into the pandemic (using 36 antibodies and 55 sera samples) were a third more predictive (46% versus 32% predicted mutations observed in the pandemic) than the experiments conducted 7 months previously (using just ten antibodies) (Fig. 3a, Extended Data Fig. 5 and 6 and Supplementary Table 6).

Despite being computed on sequences available more than 17 months earlier, EVEscape was as good as or better than the latest DMS scans at anticipating pandemic variation (50% versus 46% predicted mutations observed, respectively, when considering the top decile of prediction) (Fig. 3a). As we considered higher-frequency mutations, EVEscape increasingly predicted a greater portion of pandemic variations than experiments (Fig. 3b) and predicted a larger fraction of mutations in VOC strains (Fig. 3c).

**Fig. 4:**
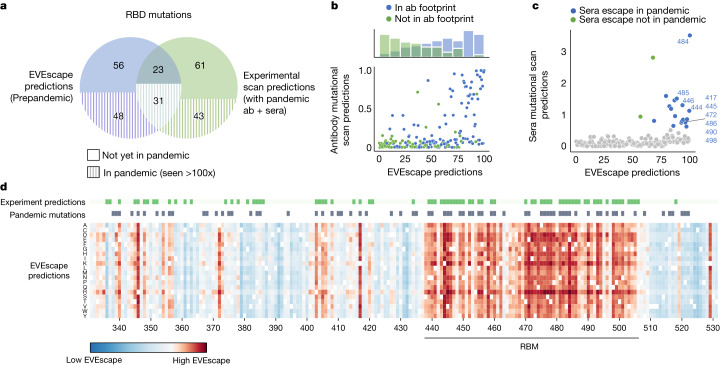
EVEscape and experiments make distinct, complementary escape predictions. **a**, Share of top decile of predicted escape mutations, predicted using EVEscape or mutational scan experiments (Bloom Set, Supplementary Table 5), seen so far more than 100 times in the pandemic. As the virus evolves further, more of the predicted escape mutations are expected to appear. **b**, RBD site-averaged EVEscape scores agree with site-averaged antibody escape experimental mutational scan measures (Bloom Set, Supplementary Table 5), with high EVEscape sites that are missing from experimental escape prediction found within known antibody footprints. Hue indicates known antibody footprints from the PDB (information that EVEscape as a prepandemic model does not use). **c**, Predicted escape mutations from experimental mutational scans (Bloom Set, Supplementary Table 5) measuring recognition by convalescent sera from patients infected with either Wuhan, Beta or Delta strains have high EVEscape scores. Sites that escape sera are coloured by whether they have occurred in the pandemic more than 1,000 times. **d**, Heatmaps illustrating the EVEscape scores of all single mutations to the Wuhan sequence of SARS-CoV-2 RBD. Top lines are sites with observed pandemic mutation frequency >100 and sites in the top 15% of DMS experimental predictions from mutational scan experiments. Source data

Discrepancies between EVEscape and experiments shed light on the complementary strengths of these approaches. EVEscape and experiments missed 43 and 48 pandemic mutations, respectively, that were predicted by the other method (Fig. 4a,d). These differences could indicate model inaccuracies, or they could reflect sparse sampling of host sera response in DMS experiments, as well as artefacts from experiments testing only the RBD domain and missing the full set of in vivo constraints. Indeed, as more antibodies were incorporated in experiments, the agreement between EVEscape and experimental predictions increased (Extended Data Fig. 6d). Most of the high EVEscape predictions that were not observed in experimental predictions were in known antibody epitopes (Fig. 4b and Extended Data Fig. 3e). By contrast, those mutations identified by the experiments that were below the threshold for EVEscape predictions were often predicted to have low fitness owing to high conservation in the alignment at those positions (Supplementary Table 6).

The consensus between EVEscape and experiments is also of interest. Agreement was especially strong for polyclonal patient sera (Supplementary Table 8); in fact, half of the top 10% of EVEscape RBD sites were sera escape sites from experiments^4–6,13,14^ (Fig. 4c). Whereas antibody mutational scans are biased towards antibodies with potential therapeutic relevance, the escape mutations from polyclonal sera are of particular interest as they depict real pandemic selection pressures in convalescent patients and are thus crucial to considerations of reinfection and vaccine design. For instance, E484, mutated in several VOCs, had the highest experimental sera binding and was the top EVEscape predicted site.

## Adapting EVEscape through its modular framework

**Extended Data Fig. 7:**
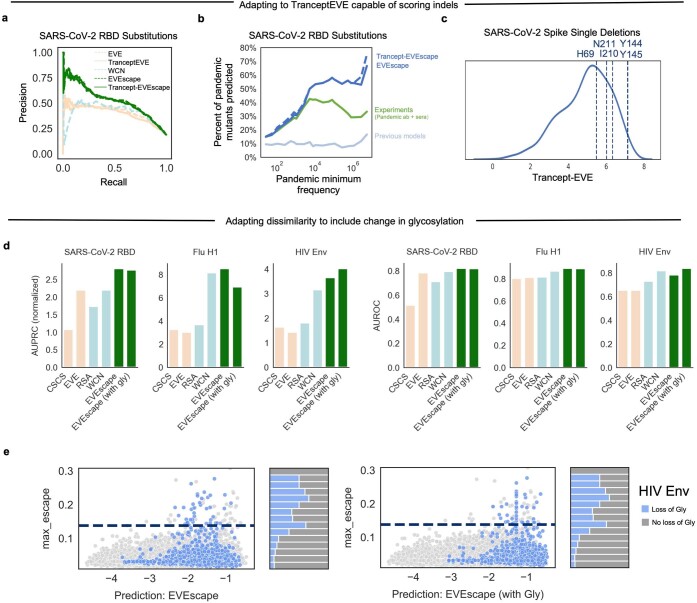
EVEscape adapts to new models: incorporating glycosylation and a transformer model of mutation fitness capable of scoring indels. a) The EVEscape fitness component can be substituted with a new generative model, Trancept-EVE^43^ that is capable of scoring substitutions as well as insertions and deletions. EVEscape using TranceptEVE as the fitness model performs equivalently to EVEscape using EVE at predicting substitutions from deep mutational scans that escape antibody binding. b) Percent of the top 10% EVEscape predicted substitutions using either EVE or TranceptEVE that were observed at different frequency thresholds during the pandemic shows that EVEscape with TranceptEVE is just as good as, or better than, EVEscape using EVE at predicting pandemic substitutions. c) Histogram of EVEscape scores (with TranceptEVE as a fitness model) for all single deletions to Spike. Single deletions seen in the pandemic more than 1000 times (vertical lines) are predicted higher than most other single deletions, especially the very frequent pandemic deletion Y144- (seen more than a million times). d) Incorporating glycosylation in EVEscape improves performance on HIV Env. Precision-Recall (with AUPRC normalized by “null” model – fraction of observed escapes) (left) and AUROC (right) of EVEscape and EVEscape+Gly models predicting DMS escape mutations for SARS-CoV-2 RBD, Flu H1, and HIV Env. e) Scatterplot of HIV Env maximum DMS escape vs. EVEscape predictions with and without glycosylation. Hue indicates mutations that cause loss of glycosylation. The majority of HIV Env escape mutations involve glycosylation loss, and EVEscape+Gly performs better on these mutations.

**Extended Data Fig. 8:**
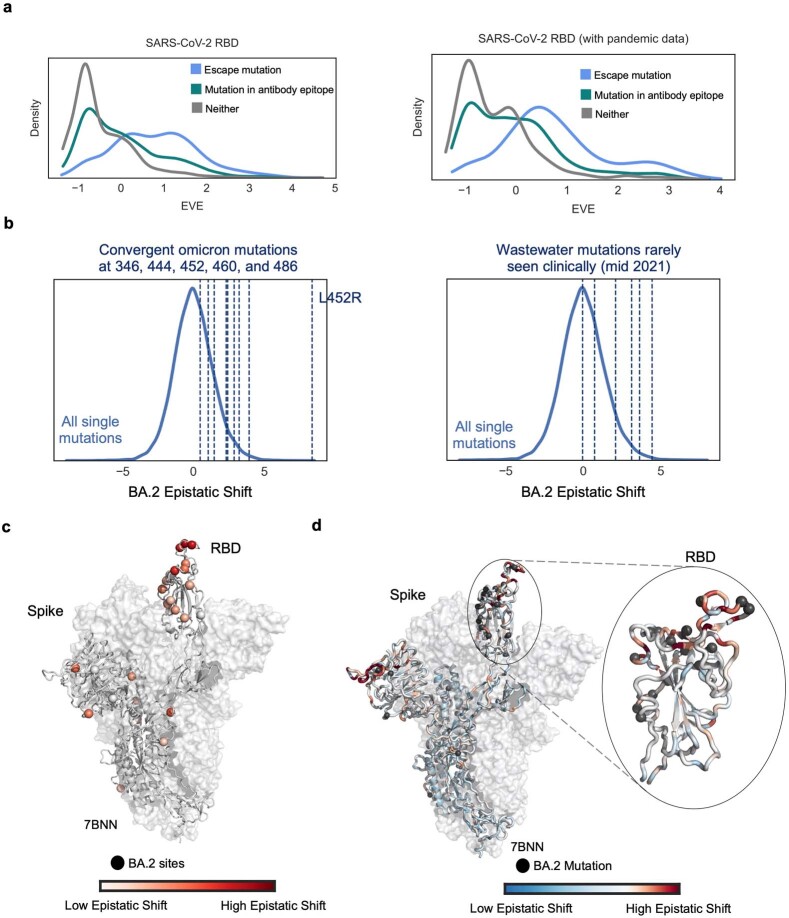
EVEscape later in a pandemic: using pandemic data and capturing epistatic shifts. a) Incorporating pandemic sequences in EVE training data results in a greater distinction between the distributions of escape and non-escape mutation EVE scores. b) Histogram of epistatic shift values between Wuhan and BA.2 strain EVE models for all single mutations, calculated as linear regression residuals. Convergent mutations that arise multiple times in Omicron lineages (mutations at sites 346, 444, 452, 460, and 486) are highlighted on the left. Wastewater mutations seen mid-2021^44^ that were rarely seen clinically in patients, and so likely epistatic, are highlighted on the right. c) Max epistatic shift magnitudes of mutations at sites mutated in BA.2 shows high epistatic shifts concentrated in the RBD. d) Large epistatic shifts for mutations on Wuhan and BA.2 strains are concentrated at sites proximal to BA.2 mutations.

The modular design of our framework facilitates its adaptability to the specific characteristics of a pandemic and to new data as they become available. To consider the effects of insertions and deletions (indels) on SARS-CoV-2 Spike immune escape, we replaced the EVE fitness component with TranceptEVE^43^, a recently developed protein large language model that has previously shown state-of-the-art performance for prediction of the effects of mutations, including indels, which both previous computational models and high-throughput experiments have been unable to capture for SARS-CoV-2. When applied to the pandemic, this model captured the most frequent single insertion and deletion, both at site 144, and each in the top decile of pandemic and random indel predictions (Extended Data Fig. 7). We also found that including glycosylation in the dissimilarity component for HIV Env, for which glycans play an important part in immune escape, improved model predictions of high-throughput experimental escape^16^ (the AUPRC increased by 10% when glycosylation was included for HIV; Extended Data Fig. 7). We also retrained EVE models with the addition of 11 million new sequences collected during the pandemic, which improved agreement with fitness DMS experiments by 20% (Extended Data Figs. 2 and 8 and Supplementary Tables 1 and 2). This model captured epistatic shifts between Wuhan and BA.2 strains, identifying changes in mutation fitness in the RBD and near BA.2 mutations, and predicting positive epistatic shifts for known convergent omicron mutations and probable epistatic wastewater mutations^44^ (Extended Data Fig. 8).

## Strain forecasting with EVEscape

A key application of an escape prediction framework is to identify circulating strains with high immune escape potential soon after their emergence, enabling the deployment of targeted vaccines and therapeutics before their spread. Although the World Health Organization seeks to identify new high-risk variants as they arise, new strains are occurring at an increasing rate, with tens of thousands of new SARS-CoV-2 strains each month now, a scale unfeasible for experimental risk assessment. To create strain-level escape predictions, we aggregated EVEscape predictions across all individual Spike mutations in a strain. We evaluated EVEscape strain predictions for their alignment with experimental measures of strain immune evasion, as well as their identification of known escape strains from pools of random sequences and from other strains observed at the same pandemic timepoint.

**Fig. 5:**
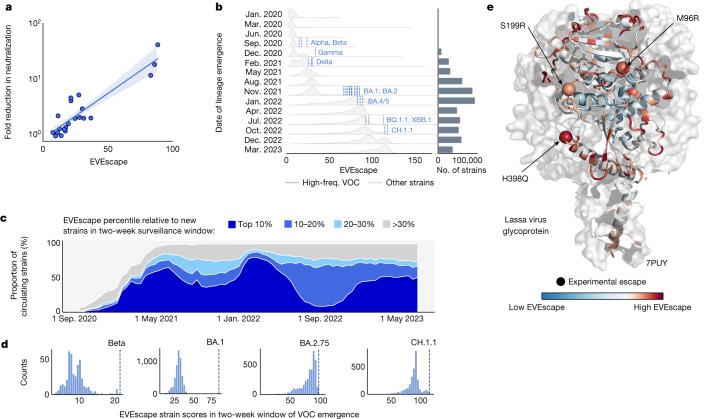
Identifying strains with high escape potential and forecasting escape for future pandemics. **a**, Prepandemic EVEscape scores computed for pandemic strains correlate with fold reduction in pseudovirus 50% neutralization titre^19^ for each strain relative to the Wuhan strain (*ρ* = 0.81, *n* = 21). Linear regression line shown with a 95% confidence interval. **b**, Distributions of newly emerging EVEscape strain (unique combination of mutations) scores for non-VOCs throughout 15 periods of the pandemic, with counts of unique new strains per period. EVEscape strain scores increased throughout the pandemic. High-frequency VOC (occurring more than 5,000 times) scores are shown as vertical lines in the first period in which each emerged; new VOCs were predicted to have higher escape scores than most strains in all previous time periods. **c**, Pandemic circulating strains are grouped according to their EVEscape decile relative to other strains emerging in the same non-overlapping two-week surveillance window. The relative prevalence of each EVEscape decile over the course of the pandemic is plotted in a stacked line-plot. More than 40% of circulating strains on average fall into the top 10% bin. Proportions do not sum to 100% as strains that emerged before the surveillance period of September 2020 to June 2023 are not included. **d**, VOCs (dotted lines) were among the highest scoring of hundreds or thousands of new strains (histograms) within their two-week window of emergence, enabling EVEscape to forecast which strains will dominate as soon as they appear after only a single observation. **e**, Site-wise maximum EVEscape scores on Lassa virus glycoprotein structure (PDB: 7PUY). We show agreement between sites of high EVEscape scores (in red) and escape mutations with experimental evidence (shown with spheres). freq., frequency. Source data

**Extended Data Fig. 9:**
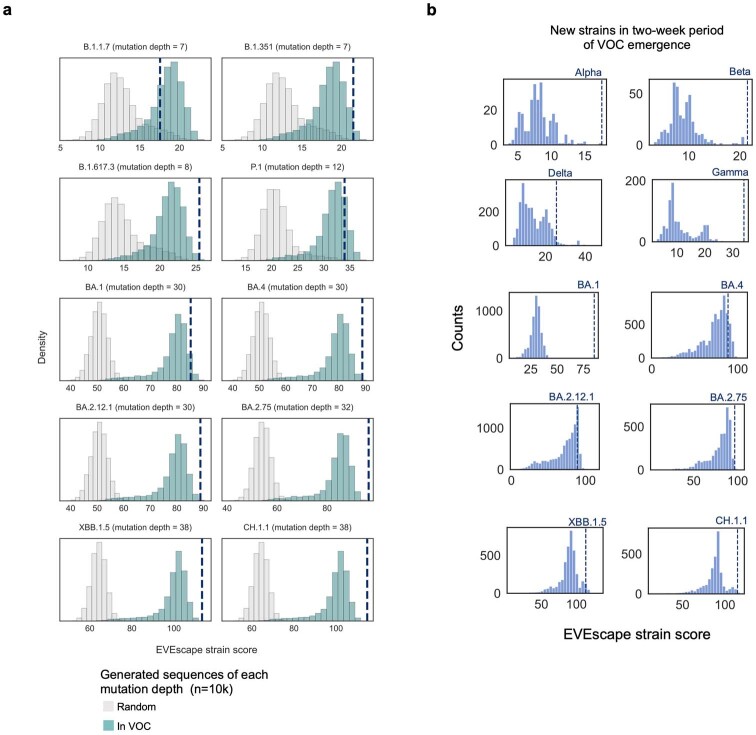
EVEscape strain forecasting. a) VOCs have high EVEscape scores compared to combinations of random mutations (sampled either from all possible single substitution mutations or from mutations previously observed in VOCs) at the same mutation depth, particularly Delta and later Omicron strains. b) VOCs are among the highest scoring new, unique strains for their two-week period of emergence using a prepandemic EVEscape model.

First, we found that prepandemic EVEscape strain scores correlated well with the results of experiments quantifying vaccinated sera neutralization of 21 strains^19^ (*ρ* = 0.81; Fig. 5a and Supplementary Table 9) and were better than those obtained with an existing computational strain-scoring method (*ρ* = 0.77)^19^, even though that method used 332 pandemic antibody-Spike structures for the prediction. Second, we found that EVEscape strain scores for VOCs were consistently higher than random sequences at the same mutational depth; in particular, the Beta, Gamma, Delta, Omicron BA.4, BA.2.12.1, BA.2.75, XBB.1.5 and CH.1.1 strain scores were in the top 1% of these generated sequence scores (Extended Data Fig. 9). EVEscape strain scores for Delta and the later Omicron VOCs were also in the top 1% against sequences composed only of mutations already known to be favourable—mutations sampled from other VOCs (Extended Data Fig. 9).

Last, we examined the ability of EVEscape to identify immune-evading strains as they emerged in the pandemic. EVEscape scores increased throughout the pandemic and were higher for more recent VOCs, reflecting their increased propensity for immune escape (Fig. 5b). Moreover, EVEscape scores for newly emerging VOCs were higher than those for almost all strains in previous time periods (Fig. 5b). Taken together, these results indicate the promise of EVEscape as an early-detection tool for picking out the most concerning variants from the large pool of available pandemic sequencing data. We therefore examine the utility of EVEscape as a tool to identify strains with high escape potential as they emerge. We classify ‘high-escape strains’ as the top decile of sequences with the highest EVEscape scores of all new and distinct strains present during a two-week surveillance window. These high-escape strains were consistently the predominant variants throughout the pandemic, constituting on average more than 40% of circulating sequences (Fig. 5c). Moreover, in the two-week windows in which the VOC strains Alpha, Beta, Gamma and Omicron BA.1 emerged, each VOC ranked first of hundreds or thousands of new strains (Fig. 5d and Extended Data Fig. 9). This demonstrates the ability of EVEscape to forecast which strains will dominate as soon as they appear after only a single observation, even as experimental testing of all emerging strains has become intractable.

To enable real-time variant escape tracking, we make monthly predictions (Supplementary Table 9) available on our website (evescape.org), with EVEscape rankings of newly occurring variants from GISAID and interactive visualizations of probable future mutations to our top predicted strains. In sum, the EVEscape model captures relative immune evasion of successful strains and can identify concerning strains from pools of random combinations of mutations as well as from their temporal peers.

## EVEscape generalizes to other viral families with pandemic potential

**Extended Data Fig. 10:**
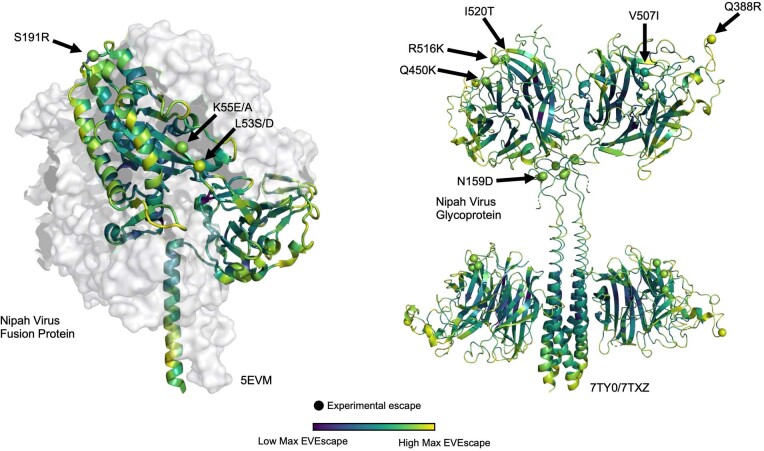
EVEscape predictions for potential pandemics. Site-maximum EVEscape scores for Nipah Virus fusion protein (left) and Glycoprotein (right) depict regions of high EVEscape scores. Known escape mutations with experimental evidence^46–50^ (little is known for this understudied virus with pandemic potential) are highlighted with spheres.

Most viruses with pandemic potential are subjected to far less surveillance and research than SARS-CoV-2. One of the main features of EVEscape is the ability to predict viral antibody escape before a pandemic—without the consequent increase in data during a pandemic—to select vaccine sequences and therapeutics most likely to provide lasting protection, to assess strains as they arise and to provide a watch list for mutations that might compromise any existing therapies. As one of the first comprehensive analyses of escape in these viruses, we applied the EVEscape methodology to predict escape mutations to the Lassa virus and Nipah virus surface proteins; these viruses cause sporadic outbreaks of Lassa haemorrhagic fever in West Africa and highly lethal Nipah virus infection outbreaks in Bangladesh, Malaysia and India. Crucially, the three mutants present in Lassa that are known to escape neutralizing antibodies^45^ were all in the top 10% of EVEscape predictions, indicating that EVEscape captures features relevant to Lassa glycoprotein antibody escape (Fig. 5e and Supplementary Table 6). EVEscape predictions also identified ten of 11 known escape mutants to Nipah antibodies^46–50^ (Extended Data Fig. 10).

Moreover, we demonstrate generalizability to influenza hemagglutinin^15^ and HIV Env^16^ using DMS evaluation (Extended Data Fig. 6). On the basis of these findings, we provide all single mutant escape predictions for these proteins (Supplementary Table 6) to inform active and continuing vaccine development efforts with the goal of mitigating future epidemic spread and morbidity.

## Discussion

One of the greatest obstacles to the development of vaccines and therapeutics to contain a viral epidemic is the high genetic diversity derived from viral mutation and recombination, especially under pressure from the host immune system. An early sense of potential escape mutations could inform vaccine and therapeutic design to better curb viral spread. Computational models can learn from the viral evolutionary record available at pandemic onset and are widely extensible to mutations and their combinations. However, new pandemic constraints (such as immunity) are unlikely to be captured. To achieve early escape prediction, EVEscape combines a model trained on historical viral evolution with a biologically informed strategy using only protein structure and biophysical constraints to anticipate the effects of immune selection. Through a retrospective analysis of the SARS-CoV-2 pandemic, we demonstrate that EVEscape forecasts pandemic escape mutations and can predict which emerging strains have high escape potential. This computational approach can preempt predictions from experiments that rely on pandemic antibodies and sera by many months while providing similar accuracy.

EVEscape provides surprisingly accurate early predictions of prevalent escape mutations but cannot anticipate all constraints unique to a new pandemic to determine the precise trajectory of viral evolution. This method will be best leveraged in synergy with experiments developed to measure immune evasion and enhanced with pandemic data as they become available. Early in a pandemic, EVEscape can predict probable escape mutations for prioritized experimental screening with the first available sera samples—validated escape mutations could be strong candidates for multivalent vaccines. EVEscape can also identify structural regions with high escape potential, so therapeutic antibody candidates with few potential escape mutants in their binding footprint may be accelerated. Later in a pandemic, EVEscape can rank emerging strains, as well as mutants on top of prevalent strains, for their escape potential, flagging concerning variants early for rapid experimental characterization and incorporation into vaccine boosters. The model could also be augmented to leverage current knowledge on virus-specific immune targeting and mutation tolerance from experimental and pandemic surveillance data. In return, our computational framework can inform this collective understanding by proposing escape variant libraries for focused experimental investigations.

EVEscape is a modular, scalable and interpretable probabilistic framework designed to predict escape mutations early in a pandemic and to identify observed strains and their mutants that are most likely to thrive in a populace with widespread preexisting immunity as the pandemic progresses. To this end, we provide EVEscape scores for all single mutation variants of SARS-CoV-2 Spike to the Wuhan strain, as well as scores for all observed strains and predictions of single mutation effects on the most concerning emerging strain backgrounds, with plans to continuously update with new strains. As the framework is generalizable across viruses, EVEscape can be used from the start for future pandemics, as well as to better understand and prepare for emerging pathogens. To further accelerate broad and effective vaccine development, we provide EVEscape mutation predictions for all single mutations to influenza, HIV, Lassa virus and Nipah virus surface proteins. Methods are provided in the Supplementary Information.

### Reporting summary

Further information on research design is available in the Nature Portfolio Reporting Summary linked to this article.

## Online content

Any methods, additional references, Nature Portfolio reporting summaries, source data, extended data, supplementary information, acknowledgements, peer review information; details of author contributions and competing interests; and statements of data and code availability are available at 10.1038/s41586-023-06617-0.

### Supplementary information

Supplementary Information Supplementary Methods.

Reporting Summary

Supplementary Table 1 Description of model inputs, including taxa of sequences in SARS-CoV-2 Spike and RBD training alignments (RBD and Spike without pandemic data are the primary alignments used throughout this paper), EVE training alignment summary statistics and PDB structures capturing diverse protein conformations used for accessibility calculations.

Supplementary Table 2 Alignments used for EVE models for Lassa virus, Nipah virus, SARS-CoV-2, HIV and influenza.

Supplementary Table 3 EVE, EVmutation and independent model mutation scores for DMS fitness experiments for SARS-CoV-2, HIV and influenza.

Supplementary Table 4 Experimental details of DMS fitness and escape experiments and EVE, EVmutation and independent model performance (Spearman correlations) for DMS fitness prediction experiments. Escape DMS data used for EVEscape validation.

Supplementary Table 5 EVEscape performance for selection of factor-specific temperature scaling.

Supplementary Table 6 EVEscape scores for all SARS-CoV-2, HIV, influenza, Lassa virus and Nipah virus mutations. Includes pandemic counts, RBD antibody class and DMS escape experiment scores used for Spike.

Supplementary Table 7 Forecasting of clinical antibody epitope escape mutations.

Supplementary Table 8 EVEscape performance on escape DMS data is generalizable across viruses and robust to antibody and sera samples. Precision-recall (with AUPRC normalized by ‘null’ model) and area under the receiver operating curve for predicting DMS escape mutations, for SARS-CoV-2 RBD, influenza H1 and HIV Env, as well as SARS-CoV-2 RBD antibody and sera stratification.

Supplementary Table 9 EVEscape scores for all SARS-CoV-2 pandemic lineages and scores for strain neutralization variants.

Supplementary File 1 Acknowledgements for all GISAID sequences.

### Source data

Source Data Fig. 2

Source Data Fig. 3

Source Data Fig. 4

Source Data Fig. 5

## Data Availability

The data analysed and generated in this study, including multiple sequence alignments used in training, single-mutant pandemic frequency data and fitness and escape DMS data used for validation, and predictions from our model are available in the Supplementary Information and at https://evescape.org/ and https://github.com/OATML-Markslab/EVEscape. All SARS-CoV-2 pandemic strain sequencing data are available through https://gisaid.org/. We acknowledge all data contributors, that is, the authors and their originating laboratories responsible for obtaining the specimens, and their submitting laboratories for generating the genetic sequence and metadata and sharing through the GISAID initiative. The evaluation of this study was based on metadata associated with 15,667,960 sequences available on GISAID up to 6 June 2023 and accessible at 10.55876/gis8.230814cp (Supplementary File 1). RBD DMS data used for model evaluation are available from https://github.com/jbloomlab/SARS2_RBD_Ab_escape_maps; a complete list of DMS data used for evaluation is available in Supplementary Table 4. We also evaluated against clinical antibody escape susceptibility data from https://covdb.stanford.edu/. We used the following Protein Data Bank (PDB) identifiers: 6VXX, 6VYB, 7CAB, 7BNN, 1RVX, 5FYL, 7TFO, 7PUY, 5EVM, 7TY0 and 7TXZ (Supplementary Table 1). Previous models of antibody escape are available from https://github.com/3BioCompBio/SpikeProSARS-CoV-2 and https://github.com/brianhie/viral-mutation. Multiple sequence alignments were constructed with sequences from https://www.uniprot.org/uniref/?facets=identity%3A1.0&query=%2A. Source data are provided with this paper.

## Code availability

The model code is available at https://github.com/OATML-Markslab/EVEscape.
