# Supplementary material for: Learning from prepandemic data to forecast viral escape
Source: Nature. 2023 Oct 11;622(7984):818–25. doi: 10.1038/s41586-023-06617-0 (PMC10599991; doi:10.1038/s41586-023-06617-0)
Supplement: Supplementary file 1 — Supplementary Methods. [file 41586_2023_6617_MOESM1_ESM.pdf]

nature portfolio

https://doi.org/10.1038/s41586-023-06617-0

Supplementary information

Learning from prepandemic data to forecast viral escape

In the format provided by the authors and unedited

Supplementary Information Methods for:

Learning from pre-pandemic data to forecast viral escape

Authors

Nicole N. Thadani[1,*], Sarah Gurev[1,2,*], Pascal Notin[3,*], Noor Youssef[1], Nathan J. Rollins[1,†], Daniel Ritter[1], Chris Sander[1,4], Yarin Gal[3], Debora S. Marks[1,4,**]

Affiliations

[1]Marks Group, Department of Systems Biology, Harvard Medical School, Boston, MA, USA
[2]Department of Electrical Engineering and Computer Science, MIT, Cambridge, MA, USA
[3]OATML Group, Department of Computer Science, University of Oxford, Oxford, UK
[4]Broad Institute of Harvard and MIT, Cambridge, MA, USA

*These authors contributed equally to this work
**Corresponding author: debbie@hms.harvard.edu

Present addresses:
†Seismic Therapeutic, Watertown, MA, USA

Supplementary Information Methods

Data acquisition:

Training Data

Multiple sequence alignments for fitness models

For each viral protein, we construct multiple sequence alignments performing 5 iterations of the profile-HMM based homology search tool jackhmmer[51] against the UniRef100 database[52]. As previously reported for EVE, DeepSequence, and EVcouplings, we generally keep sequences that align to at least 50% of the target sequence and columns with at least 70% coverage, except in the case of SARS-CoV-2 Spike where we use lower column coverage as needed (30-70%) to maximally cover experimental positions and significant pandemic sites[20–22].

For our pre-pandemic (pre-2020) alignment used as the primary model throughout this paper, we remove pandemic sequences using the "date of creation" variable from UniRef. We optimize search depth to maximize sequence coverage and the effective number of sequences (Neff) included after re-weighting similar protein sequences in the alignment within a Hamming distance cutoff (theta) of 0.01. To select sequence depth, we prioritized alignments with coverage >0.7L and Neff/L>1, or if this was not attainable, relaxed the requirements for Neff/L (Supplementary Table 1).

Alignments with pandemic sequences

We construct an "evolutionary alignment" with non-SARS-CoV-2 sequences as described above using jackhmmer (with at least 50% sequence coverage, at least 30% column coverage, and theta of 0.01). We extract the full sequences pulled into the jackhammer alignment and re-align the sequences using super5[53], then remove gapped positions relative to the Wuhan sequence. We also construct a "pandemic alignment" with all unique Spike sequences (with count >100) seen up until 11/27/21 (when BA.2 first appeared in GISAID). We then concatenate that "pandemic alignment" with the "evolutionary alignment" to create the final alignment.

Protein structures for accessibility calculation

For each viral surface protein, we selected crystal structures representing known structural states available to B-cell and antibody interactions (extracellular conformations) (Supplementary Table 1). All heteroatoms and protein chains not part of the multimeric viral surface protein were removed.

Evaluation data

Antibody footprints

To identify known antibody footprints of viral surface proteins in the RCSB PDB[54], we queried the database with the protein name and the word "antibody" and required that the source organism contain both "Homo sapiens" and the given virus name. Then for each structure we identified antibody and viral protein polymer entities and computed the antibody footprint as any residue with any atom within 3.5 angstroms of the antibody. Finally, we mapped footprints to the target viral protein sequence by using SIFTS to renumber all hits according to a UniProt ID, then used a MUSCLE multiple sequence alignment of the different UniProt sequences to map those hits to the target viral protein sequence. We use this same method to identify antibody footprints for specific clinical antibodies. For experimental evidence of clinical antibody escape

susceptibility, we used the Stanford Coronavirus Antiviral & Resistance Database (CoV-RDB) susceptibility summary for monoclonal antibodies under emergency use authorization.[55].

Deep mutational scans

We benchmark our models on a series of viral protein deep mutational scans[2–16,25–32] (Supplemental Tables 3, 4, 6). For each viral mutational scan, we select the variable or variables of protein fitness or antibody escape treated as primary in the publications. For mutants where the result is provided as residue frequencies observed at a given site (such as results expressed as preferences and processed by dms_tools2), we normalize the data at each site by dividing by the value of the wild-type residue. For the HIV analysis, we exclude antibody VRC34.01 due to its large spread of escape mutation distal to the epitope[56]. For SARS-CoV-2 RBD, we use only antibodies/sera escape data from the Wuhan sequence for our primary results. We also utilize data provided about the antibodies tested for the SARS-CoV-2 escape DMS studies, including the class of each antibody as well as the SARS-CoV-2 neutralization potency and Sarbecovirus binding breadth[8]. We use the RBD dimeric ACE2 binding and expression DMS data for analysis[30].

Pandemic sequencing data

We downloaded data on Spike variants and their deposit dates in the Global Initiative on Sharing All Influenza Data (GISAID) EpiCoV project database (www.gisaid.org)[57] on 6/12/23. We further processed this data to get counts of combinations of mutations, the date of emergence, and PANGO lineage, as well as to get the month of emergence and count for each single mutation in Spike. We also downloaded consensus mutations for each PANGO lineage on 6/21/23 Covid-19 CG[58].

Lassa virus and Nipah virus antibody escape data

We aggregated data on single mutations resulting in escape from known Lassa and Nipah virus antibodies from literature studies with experimentally determined reduction in antibody binding, reduction in antibody neutralization, or emergence in growth selection experiments[45–50].

Epistasis mutation sets

Our convergent omicron mutation set is defining mutations in Omicron lineages at sites 346, 444, 452, 460, and 486. This set is: L452R, N460K, F486V, K444N, L452M, F486I, R346T, F490S, K444M, K444T.

Our wastewater mutation set is the set of mutations from Smyth et al.[44], which are mutations that were frequent in wastewater, but had rarely been seen clinically (pre-Omicron, mid 2021), so may be likely epistatic. This set is: Q493K, Q498Y, Q498H, T572N, H519N, H519Q.

Strain Neutralization data

We download neutralization data from Beguir et al.[19], which contains the observed 50% pseudovirus neutralization titer ($pVNT_{50}$) for 21 SARS-CoV-2 S protein variants. The $pVNT_{50}$ reduction is relative to Wuhan. Neutralization is measured for n ≥ 12 sera collected after primary 2-dose vaccination by the Pfizer BioNTech vaccine (BNT162b2) and assessed against vesicular stomatitis virus (VSV)-based pseudoviruses with each S protein variant.

Modeling approach:

Overarching framework

We express the probability of a single amino acid substitution to lead to immune escape as the product of three conditional probabilities (Fig. 1a):

$$
\begin{aligned}
&\boldsymbol{P}(Mutation\ escapes\ immunity) \\
&\quad = \boldsymbol{P}(Mutation\ maintains\ fitness) * \boldsymbol{P}(Mutation\ accessible\ to\ antibody \mid fit) \\
&\quad * \boldsymbol{P}(Mutation\ disrupts\ antibody\ binding \mid fit, accessible)
\end{aligned}
$$

The EVEscape index estimates the log likelihood of escape as per the above equation. The fitness factor is obtained via a deep generative model for fitness prediction, while the accessibility and dissimilarity factors are features derived respectively from the known 3D structures for the viral protein and chemical characteristics of the amino acids involved in the mutation compared to the wild-type (see below for details).
Once selected, each factor is standardized and fed into a temperature scaled logistic function:

$$
\begin{aligned}
&\boldsymbol{P}(Mutation\ escapes\ immunity) \\
&\quad = logistic\left(\frac{1}{T_{fitness}} * standardize\left(F_{fitness}\right)\right) \\
&\quad * logistic\left(\frac{1}{T_{accessibility}} * standardize\left(F_{accessibility}\right)\right) \\
&\quad * logistic\left(\frac{1}{T_{dissimilarity}} * standardize\left(F_{dissimilarity}\right)\right)
\end{aligned}
$$

where the standardize(.) operator corresponds to standard scaling. We then take the log transform of the product of the 3 terms to obtain the final EVEscape scores.

Scaling

Factor-specific temperature scaling helps recalibrate probability estimates for each term. We provide our hyperparameter grid search of these temperature hyperparameters across viruses in Supplementary Table 5, examining versions of the model where we either include or do not include glycosylation in the dissimilarity term. We run this grid search using AUROCs to compare model predictions to experimental escape DMS predictions for 3 viral proteins (Influenza H1[15], HIV Env[16], and SARS-CoV-2 Spike RBD[2–11,13,14]). We find that the fitness and accessibility components are already properly calibrated ($T_{fitness}$ = $T_{accessibility}$ = 1.0), while the dissimilarity component benefits from being slightly rescaled ($T_{dissimilarity}$ = 2.0). The optimal values selected for the three temperature hyperparameters yield strong performance across the three viruses, suggesting they are generalizable to other viruses.

Fitness metric

Observed viral protein sequences reflect evolution under selection constraints for functional and infectious viruses. Generative sequence models express the probability that a sequence x would be generated by this process as $p(x|\theta)$, where the parameters θ capture the constraints describing functional variants. A generative model trained on observed viral protein variants can

then be used to estimate the relative plausibility of a given mutant sequence as compared to wild-type by using the log ratio of sequence likelihoods as a heuristic:

$$log\frac{p(x^{mutant}|\theta)}{p(x^{wildtype}|\theta)}$$

EVE

EVE (Evolutionary model of Variant Effects)[20] is a Bayesian variational autoencoder (VAE)[59], capable of capturing intricate higher-order interactions across sequence positions. The architecture consists of a symmetric encoder and decoder architecture, each with 3 layers with 2,000-1,000-300 and 300-1,000-2,000 units respectively, as well as a 50-dimensional latent space (Extended Data Fig. 1). As generative models, VAEs can learn a complex distribution of the high-dimensional data on which they are trained, in our case, sequences from a specific protein family. More formally, for a protein family **p**, we learn a distribution $P(\mathbf{s}|\boldsymbol{\theta}_\mathbf{p})$, where **s** is a fixed-length amino-acid sequence and $\boldsymbol{\theta}_\mathbf{p}$ are model parameters associated with that protein family. Variational Autoencoders operate under the assumption that the data **s** are generated from a latent variable **z.** They model the conditional distribution $P(\mathbf{s}|\mathbf{z},\boldsymbol{\theta}_\mathbf{p})$ with a neural network (also known as the "decoder", with parameters $\boldsymbol{\theta}_\mathbf{p}$), and leverage amortized inference to model the approximate posterior distribution $q(\mathbf{z}|\mathbf{s},\boldsymbol{\varphi}_\mathbf{p})$ with another neural network (known as the "encoder", with parameters $\boldsymbol{\varphi}_\mathbf{p}$). Lastly, following Riesselman et al.[22] and Frazer et al.[20], our Bayesian VAE departs from the standard VAE architecture by learning a fully-factorized Gaussian distribution over the decoder weights $\boldsymbol{\theta}_\mathbf{p}$.

The fitness of a given protein sequence is then quantified via the log likelihood ratio of the mutated sequence *x* over that of the reference wild-type sequence *w*. Since an exact computation of the log likelihood of a sequence is intractable, we approximate it with the Evidence Lower Bound (ELBO) used to optimize the VAE:

$$E_{EVE}(x) = log\frac{p(x|\theta)}{p(w|\theta)} \sim ELBO(x) - ELBO(w)$$

The ELBO term itself is estimated via Monte Carlo sampling, using 20k samples from the approximate posterior distribution. These approximations have been shown to provide strong results in practice[20]. Results are obtained by ensembling scores from 5 independently trained EVE models with different random seeds. Note that this is the negative of the evolutionary index score outputted by the EVE model.

We train the different models following the procedure from the original EVE paper (see Frazer et al.[20], Supplementary Section 3.2), using similarly-sized EVE models and with the same training hyperparameters. The only difference in our training procedure is that we slightly relax the constraint on minimum column coverage for sequences in the training MSAs (50% instead of 70%) as it led to superior fitness prediction performance in our hyperparameter tuning analyses for the different viruses modeled in this work.

TranceptEVE

In experiments aimed at illustrating the modularity of the EVEscape framework we leverage TranceptEVE, a recently developed protein language model with state-of-the-art performance

for mutation effects predictions[43]. TranceptEVE is itself based off of two key components: 1) Tranception[60], a family-agnostic autoregressive transformer trained on a large quantity of unaligned protein sequences from Uniref100[52] from February 2022. 2) A family-specific EVE model that is trained to score sequences for a family of interest, and which acts as a prior distribution over amino acids at each sequence position. The predicted fitness for a given sequence is then obtained as a weighted average of the log likelihood assigned by these two components – the weights depending on the depth of the alignment used to train the underlying EVE model (deeper alignments implying a larger weight assigned to the EVE log likelihood). For the experiments conducted in this work, we use the same ensemble of 5 EVE models as described above, as well as the large Tranception model checkpoint (~700M model parameters) made available in Notin et al.[60] which was trained on Uniref100 (see details of the training procedure in the corresponding paper in Appendix B.3).

Accessibility metric

Surface accessibility plays a key role in identifying where antibodies are most likely to contact a protein. While relative solvent accessibility (RSA) and weighted contact number (WCN) both reflect features of accessibility, we selected WCN as this metric also captures protrusion from the core structure that corresponds with where antibodies are known to bind proteins[33,61–63] (Supplementary Table 1).

Calculating weighted contact number

We computed weighted contact numbers[33] for each residue from structure as the sum of the square of the reciprocal distance between residue i and all other residues in the full protein (i.e., the full Spike trimer for SARS-CoV-2):

$$WCN_i = \sum_{j \neq i} \frac{1}{{r_{ij}}^2}$$

where r_{ij} is the distance between the geometric centers of the residue i and residue j side chains. Weighted contact number, beyond capturing surface accessibility, captures protrusion from the core structure and conformational flexibility[33,61–63]. By using squared distance, this value focuses on the degree of local interaction, and acts as a measure of exposure to the local environment that would permit antibody binding. It is both a simple and fast metric. We impute missing values in WCN due to gaps in the protein structure using the mean of WCN values of the residues preceding and following the gap. We use the negative weighted contact number.

RSA

We also explored RSA as a potential accessibility metric. To do so, we first computed accessible surface area based on hypothetical exposure to solvent water molecules using DSSP[64]. To calculate relative accessible surface area (RSA), we divided accessible surface area by the residue maximum accessibilities determined in Sander et al[65]. We impute missing values in RSA due to gaps in the protein structure by using the mean of RSA values of the residues preceding and following the gap (counting residues adjacent to the gap with RSA values>1 as part of the gap).

Aggregating across structures:

When computing antibody-binding likelihood metrics across different structural conformations (i.e., both open and closed structures for SARS-CoV-2 Spike) we used the maximum negative weighted contact numbers.

Dissimilarity metric

To predict the likelihood of a given mutation displacing an antibody interaction, we used a charge-hydrophobicity based measure of functional dissimilarity between the wild-type residue and the mutation residue. These are chosen as properties known to impact protein-protein interactions[34,66]. We compare our metric to individual chemical properties, substitution matrices, and the distance in the latent space of a VAE. We also experiment with incorporating glycosylation in our dissimilarity metric.

Charge-hydrophobicity

To compute a combined charge-hydrophobicity dissimilarity index, we standard-scaled the charge and hydrophobicity differences and then took the sum of the scaled differences. We use the Eisenberg-Weiss hydrophobicity consensus scale[67] and amino acid charge (as 1/0/-1) at physiological pH.

Chemical properties

We compared our metric to differences in residue size (side-chain mass), hydrophobicity, and charge.

Substitution Matrices

We compared our metric to the BLOSUM62[68] matrix after dropping the null transition diagonal values.

Latent space distances

We also compared our metric to a metric of mutation distance learned by the EVE variational autoencoder. We calculated the L1 distance between the encoded representations of the wild-type viral protein sequence and a given single-mutation sequence in the latent space of the model, inspired by a similar approach first introduced by Hie et al.[42]

Glycosylation

We developed a version of our model considering glycosylation loss as a contributor to dissimilarity. While addition of glycosylation is also important for escape[69–72], we focus here on loss of glycosylation for simplicity. In this version, we maximize the charge-hydrophobicity dissimilarity term if a mutation is likely to result in loss of a surface N-glycan site. We identified surface N-glycan sites as NxS/T sequons (where x is any amino acid except proline) with the N residue having an RSA>0.2. We consider that a mutation is likely to result in loss of glycosylation if the N or S/T is lost.

We note that this can be an important factor for real-world escape even when some DMS experiments do not reflect the escape impacts of glycosylation loss, as is the case for SARS-CoV-2 experiments that use yeast display, with glycans different than in mammalian cells[3]. For HIV on the other hand, a significant portion of escape mutations from DMS experiments are a result of escape effects of glycan gains and loss[16]. In the limited HIV Env dataset examining 8 antibodies, 50% of all escape mutations are likely due to removal of a glycan[16]. SARS-CoV-2 Spike (22 glycosylation sites) and Flu H1 (up to 11 glycosylation sites) are also much less extensively glycosylated than HIV Env (up to 30 glycosylation sites).

Imputing missing data

We impute missing values of features in EVEscape using the mean value of the feature across the target protein.

Insertions and Deletions

Scores for indels, important for SARS-CoV-2 and other virus[73], utilize tranceptEVE as the fitness component, negative weighted contact number as the accessibility component, and a maximized dissimilarity component score.

Strain-level EVEscape predictions

To compute strain-level EVEscape predictions, we use a fitness component calculated for the full strain sequence, while accessibility and dissimilarity components are summations of the single-mutation scores for each mutation in the strain. To normalize fitness scores across mutational depths, we take the percentile of the EVE score relative to the EVE scores of 10,000 sequences at the same mutational depth, randomly generated by sampling single mutations seen over 100 times in GISAID by January 2021. We then standard scale the percentile fitness score relative to the percentile EVE scores of 10,000 randomly generated sequences composed of between 2 and 50 completely random single mutations. As in the mutation-level EVEscape calculation, accessibility and dissimilarity components are standard-scaled against all possible single mutations to Spike. Standard-scaled components are then adjusted to have only positive values by adding the absolute value of the minimum scaled score for that component. Finally, we aggregate across combinations of mutations by summing the log transform of the temperature scaled logistic function of each of the three components for each mutation (so, dissimilarity and accessibility scores are additive across mutations in the strain while the fitness score is a full strain score multiplied by the number of mutations in the strain). Strain-level EVEscape scores can be further adapted, i.e., with alternative scaling techniques or learning how to combine epitope-level scores.

Evaluation approach:

Comparison to functional assays

We compared model predictions to continuous experimental metrics of viral function using spearman's rank correlation coefficient as our main evaluation metric, as previously described[21,22].

Comparison to escape DMS

Data processing

As escape data is noisy at levels of low escape and a relatively low fraction of mutants exhibit escape, we chose to treat the escape outcome variable as binary. We selected a threshold for escape by fitting a gamma distribution to the data (combined across all screened antibodies and sera) and selecting the threshold corresponding to a 5% false discovery rate[16]. As the number of antibodies tested for RBD is much higher than for Flu and HIV, we bootstrapped the RBD data selecting 8 antibodies 1000 times and fitting a gamma distribution to these samples, then selected the average 5% false discovery rate threshold. As these thresholds are subject to our choice of a false discovery rate, we also plot performance for a range of thresholds (Extended Data Fig. 6). We identified a mutant as "escape" if its maximum escape value across any antibody tested exceeded the threshold — so a mutation for RBD is "escape" if it exceeds the threshold for any antibodies/sera in the Bloom or the Xie datasets (Supplementary Table 4, 6). We use thresholds of 0.57 for Bloom RBD, 0.9 for Xie RBD, 0.054 for Flu, and 0.138 for HIV to make model comparisons; mutations designated as escape by these experimental thresholds are almost all within 5Å of the antibody they escape (Extended Data Fig. 6). Note that the downloaded RBD escape datasets were already filtered using thresholds on expression and ACE2 binding of -1 and -2.35, respectively[74].

To define a site-wise escape value, we averaged across the maximum escape values for each mutant at the site. For the antibody RBD DMS data, we define the antibody class of each mutation/site by determining the maximum number of antibodies for a given class that escape that mutation/site (Supplementary Table 6).

As the scales are different for the Bloom and Xie datasets, we focus on the original Bloom RBD DMS data when we need to consider the top fraction of escape mutations. We examine performance on Flu and HIV as a secondary analysis to confirm generalizability, as fewer antibodies have been tested and the distribution of these antibodies does not reflect known immunodominant domains.

Metrics

To compare computational model performance in classifying escape mutants, we computed two metrics. We consider area under the receiver operating curve (AUROC) and area under the precision-recall curve (AUPRC). A key feature of an escape mutant predictor is the quality of its positive 'escape' predictions, as in practice, the positive predictive value will influence costly experimental screening efforts and selection of a limited number of variants for vaccine incorporation. To reflect this, we focus on the area under the precision-recall curve (AUPRC) as a performance metric (reported relative to the AUPRC of a "null" model), although other measures of overall statistical performance (e.g., AUROC) are provided in supplementary information.

AUROC summarizes the tradeoff between true positives and false positives over a range of thresholds on the continuous model prediction score but is overly permissive in cases of imbalanced datasets–-although still suitable for assessing relative performance. The AUPRC metric summarizes the tradeoff between capturing all escape mutants (recall) and not incorrectly predicting escape mutants (precision). This approach is suitable for evaluating classification of imbalanced datasets but penalizes false positive predictions. In the case of escape predictors, false positive predictions may be due to insufficient sampling of the human antibody repertoire against the virus of interest, so this penalization is potentially too stringent. We normalize AUPRC by the "null" precision model AUPRC, which is equivalent to the fraction of escapes observed in the mutations experimentally screened. Therefore, AUPRC values are not comparable between viral proteins or subsets of DMS datasets with different fractions of escape mutations. The fraction of observed escapes in the DMS experiments are 0.17 for Bloom antibodies, 0.06 for Xie antibodies, and 0.003 for Bloom sera, as well as 0.19 for all the RBD data, 0.015 for Flu, and 0.006 for HIV – Flu and HIV data examined far fewer antibody samples (Supplementary Table 6).

Comparison to known antibody footprints

We also evaluated the model's ability to predict sites of antibody binding, as quantified by looking at antibody footprints in the RCSB PDB within a minimum all-atom distance of 3.5Å. Note that this is not information that is available to the model during training.

Comparison to pandemic data

Data Processing

We evaluate the model against occurrence of single mutations and strains in GISAID. In determining the set of Spike mutations to compare EVEscape scores to GISAID data, we consider only those mutations that are a single RNA nucleotide mutation distance from Wuhan. Variants are marked as high frequency VOCs if their count is greater than 5,000 and it occurs in the first time period (pandemic divided into 12 periods) that any strain of that PANGO lineage appears. We define PANGO lineages for the VOCs by the nonsynonymous Spike consensus mutations for that strain from COVID-19 CG that occur in greater than 70% of strain sequences, ignoring insertions and deletions. Number of occurrences in the pandemic is defined by raw counts of GISAID records with a given substitution or set of substitutions.

Metrics

We calculate the fraction of predicted mutations (top 10%) seen in the pandemic over 100 times. We expect to see an increase in this fraction over the course of the pandemic, as more variants are observed and adaptive immune pressure increases with a growing vaccinated or previously infected population. We also calculate for each observed pandemic frequency minimum threshold, the percentage of pandemic mutants seen above that observed threshold that are predicted in the top 10%. We do not expect all pandemic mutants to be captured in the top 10% of predictions, because not all pandemic mutants are related to escape. Even amongst very frequent pandemic mutations mostly present in Variants of Concern, which we expect to be more enriched for high escape potential, we do not expect all these mutations to be related to escape as some instead influence ACE2 binding or structural changes. To evaluate strain scores, we calculate the number of strains (and the corresponding percentile) that would need

to be tested to have detected selected VOCs from all new strains in the two-week window they emerged. Unique new strains are defined by unique sets of Spike substitution mutations seen at least twice in the two-week window.

Escape within clinical antibody epitopes

We look at EVEscape predictions in the footprints (within 3.5Å) of six different clinical antibody epitopes. We then notate which of these mutations have already occurred in the pandemic (observed more than 10,000 times) and which have experimental evidence of escape for those clinical antibodies as seen in CoV-RDB[55]. We list all possible mutations, not just those a single nucleotide distance from Wuhan.

Comparison to strain neutralization

We show spearman correlation with experimental strain neutralization data as well as the linear regression line shown with a 95% confidence interval. EVEscape scores for these strains are calculated based on the mutations used in the experiment for each strain, ignoring indels. We convert percent reduction in neutralization (x) to fold reduction (1/1-x).

Regional Enrichment

We examine the distribution of EVEscape predictions throughout the Spike protein and, within the RBD, between the known footprints of different antibody classes from Barnes et al.[75] We analyze enrichment of regions by comparing the average EVEscape score for the region to a distribution of the average EVEscape score of random regions. For comparison to full Spike, we compare to the scores of 500 random contiguous regions (of the same length as the region of interest) within Spike. For comparison to RBD, we compare to scores of 100 contiguous regions, using the full Spike model. We similarly compare scores of known neutralizing subregions to random regions in their respective full regions. We also compare enrichment of number of sites in the top 15% of EVEscape scores in each region relative to the length of the region. We consider the regions: NTD (sequence positions 14 - 306), RBD (319 - 542), S1* (543 - 685), and S2 (686 - 1273), where S1* refers to the region in S1 between RBD and S2. NTD and RBD are enriched in antibody sites. We also calculate the mutational tolerance of each region, the average EVE fitness score.

Epistasis

We analyze epistasis by comparing EVE scores on a Wuhan full Spike model (using a pre-pandemic alignment) and on an omicron (BA.2) full Spike model (using an alignment with data up to BA.2). The BA.2 epistatic shift is the Wuhan linear regression residual for a model fit to the two sets of EVE scores for all single mutations to full Spike. We compare the epistatic shift of two subsets of mutations, convergent omicron mutations and wastewater mutations[44], to the full set of single mutations to full Spike. We also analyze the locations of the maximum epistatic shift, in relation to the Spike structure and to the set of sites mutated within BA.2.

Comparison to other computational models

We compare published SARS-CoV-2 RBD and Spike models predictions[18,19,37,42] using metrics from above relevant to the intended purpose of each model (fitness or escape of either single mutations, sites, or strains).

References

51. Johnson, L. S., Eddy, S. R. & Portugaly, E. Hidden Markov model speed heuristic and iterative HMM search procedure. *BMC Bioinformatics* **11**, 431 (2010).
52. Suzek, B. E., Huang, H., McGarvey, P., Mazumder, R. & Wu, C. H. UniRef: comprehensive and non-redundant UniProt reference clusters. *Bioinformatics* **23**, 1282–1288 (2007).
53. Edgar, R. C. Muscle5: High-accuracy alignment ensembles enable unbiased assessments of sequence homology and phylogeny. *Nat. Commun.* **13**, 6968 (2022).
54. Berman, H. M. *et al.* The Protein Data Bank. *Nucleic Acids Res.* **28**, 235–242 (2000).
55. Tzou, P. L., Tao, K., Pond, S. L. K. & Shafer, R. W. Coronavirus Resistance Database (CoV-RDB): SARS-CoV-2 susceptibility to monoclonal antibodies, convalescent plasma, and plasma from vaccinated persons. *PLoS One* **17**, e0261045 (2022).
56. Dingens, A. S. *et al.* Complete functional mapping of infection- and vaccine-elicited antibodies against the fusion peptide of HIV. *PLoS Pathog.* **14**, e1007159 (2018).
57. Khare, S. *et al.* GISAID's role in pandemic response. *China CDC Wkly* **3**, 1049–1051 (2021).
58. Chen, A. T., Altschuler, K., Zhan, S. H., Chan, Y. A. & Deverman, B. E. COVID-19 CG enables SARS-CoV-2 mutation and lineage tracking by locations and dates of interest. *Elife* **10**, (2021).
59. Kingma, D. P. & Welling, M. Auto-Encoding Variational Bayes. *arXiv [stat.ML]* (2013).
60. Notin, P. *et al.* Tranception: protein fitness prediction with autoregressive transformers and inference-time retrieval. *arXiv [cs.LG]* (2022).
61. Haste Andersen, P., Nielsen, M. & Lund, O. Prediction of residues in discontinuous B-cell epitopes using protein 3D structures. *Protein Sci.* **15**, 2558–2567 (2006).
62. Thornton, J. M., Edwards, M. S., Taylor, W. R. & Barlow, D. J. Location of "continuous" antigenic determinants in the protruding regions of proteins. *EMBO J.* **5**, 409–413 (1986).
63. Novotný, J. *et al.* Antigenic determinants in proteins coincide with surface regions accessible to large probes (antibody domains). *Proc. Natl. Acad. Sci. U. S. A.* **83**, 226–230 (1986).
64. Kabsch, W. & Sander, C. Dictionary of protein secondary structure: pattern recognition of hydrogen-bonded and geometrical features. *Biopolymers* **22**, 2577–2637 (1983).
65. Rost, B. & Sander, C. Conservation and prediction of solvent accessibility in protein families. *Proteins* **20**, 216–226 (1994).
66. Kringelum, J. V., Nielsen, M., Padkjær, S. B. & Lund, O. Structural analysis of B-cell epitopes in antibody:protein complexes. *Mol. Immunol.* **53**, 24–34 (2013).
67. Eisenberg, D., Weiss, R. M. & Terwilliger, T. C. The hydrophobic moment detects periodicity in protein hydrophobicity. *Proc. Natl. Acad. Sci. U. S. A.* **81**, 140–144 (1984).
68. Henikoff, S. & Henikoff, J. G. Amino acid substitution matrices from protein blocks. *Proc. Natl. Acad. Sci. U. S. A.* **89**, 10915–10919 (1992).
69. Moore, P. L. *et al.* Evolution of an HIV glycan-dependent broadly neutralizing antibody epitope through immune escape. *Nat. Med.* **18**, 1688–1692 (2012).
70. Wei, X. *et al.* Antibody neutralization and escape by HIV-1. *Nature* **422**, 307–312 (2003).
71. Das, S. R. *et al.* Fitness costs limit influenza A virus hemagglutinin glycosylation as an immune evasion strategy. *Proc. Natl. Acad. Sci. U. S. A.* **108**, E1417-22 (2011).

72. Li, Y. *et al.* The importance of glycans of viral and host proteins in enveloped virus infection. *Front. Immunol.* **12**, (2021).
73. Rao, R. S. P. *et al.* Evolutionary Dynamics of Indels in SARS-CoV-2 Spike Glycoprotein. *Evol. Bioinform. Online* **17**, 11769343211064616 (2021).
74. Greaney, A. J., Starr, T. N. & Bloom, J. D. An antibody-escape estimator for mutations to the SARS-CoV-2 receptor-binding domain. *Virus Evol.* **8**, veac021 (2022).
75. Barnes, C. O. *et al.* SARS-CoV-2 neutralizing antibody structures inform therapeutic strategies. *Nature* **588**, 682–687 (2020).
