# Supplementary material for: Learning from prepandemic data to forecast viral escape
Source: Nature. 2023 Oct 11;622(7984):818–25. doi: 10.1038/s41586-023-06617-0 (PMC10599991; doi:10.1038/s41586-023-06617-0)
Supplement: Supplementary file 2 — Reporting Summary [file 41586_2023_6617_MOESM2_ESM.pdf]

Corresponding author(s): Debora Marks

Last updated by author(s): Jul 27, 2023

Reporting Summary

Nature Portfolio wishes to improve the reproducibility of the work that we publish. This form provides structure for consistency and transparency in reporting. For further information on Nature Portfolio policies, see our Editorial Policies and the Editorial Policy Checklist.

Statistics

For all statistical analyses, confirm that the following items are present in the figure legend, table legend, main text, or Methods section.

n/a	Confirmed	
☐	☒	The exact sample size (*n*) for each experimental group/condition, given as a discrete number and unit of measurement
☒	☐	A statement on whether measurements were taken from distinct samples or whether the same sample was measured repeatedly
☒	☐	The statistical test(s) used AND whether they are one- or two-sided<br>*Only common tests should be described solely by name; describe more complex techniques in the Methods section.*
☐	☒	A description of all covariates tested
☒	☐	A description of any assumptions or corrections, such as tests of normality and adjustment for multiple comparisons
☐	☒	A full description of the statistical parameters including central tendency (e.g. means) or other basic estimates (e.g. regression coefficient) AND variation (e.g. standard deviation) or associated estimates of uncertainty (e.g. confidence intervals)
☒	☐	For null hypothesis testing, the test statistic (e.g. *F*, *t*, *r*) with confidence intervals, effect sizes, degrees of freedom and *P* value noted<br>*Give P values as exact values whenever suitable.*
☐	☒	For Bayesian analysis, information on the choice of priors and Markov chain Monte Carlo settings
☐	☒	For hierarchical and complex designs, identification of the appropriate level for tests and full reporting of outcomes
☐	☒	Estimates of effect sizes (e.g. Cohen's *d*, Pearson's *r*), indicating how they were calculated

Our web collection on statistics for biologists contains articles on many of the points above.

Software and code

Policy information about availability of computer code

Data collection	Multiple sequence alignments were obtained using HMMER (3.1.b2) and MUSCLE (5.1). RSA values were obtained using DSSP (4.1.1).
Data analysis	All code is available at https://github.com/OATML-Markslab/EVEscape.

For manuscripts utilizing custom algorithms or software that are central to the research but not yet described in published literature, software must be made available to editors and reviewers. We strongly encourage code deposition in a community repository (e.g. GitHub). See the Nature Portfolio guidelines for submitting code & software for further information.

Data

Policy information about availability of data

All manuscripts must include a data availability statement. This statement should provide the following information, where applicable:

- Accession codes, unique identifiers, or web links for publicly available datasets
- A description of any restrictions on data availability
- For clinical datasets or third party data, please ensure that the statement adheres to our policy

The data analyzed and generated in this study, including multiple sequence alignments used in training, single mutant pandemic frequency data and fitness and escape deep mutational scanning data used for validation, and predictions from our model, are available in Supplementary Information, and at https://evescape.org/ and https://github.com/OATML-Markslab/EVEscape. All SARS-CoV-2 pandemic strain sequencing data is available through https://gisaid.org/. We acknowledge the authors and originating and submitting laboratories of the sequences from GISAID for sharing sequencing data (detailed acknowledgements in

Supplementary Table 10). RBD deep mutational scanning data is available from https://github.com/jbloomlab/SARS2_RBD_Ab_escape_maps – a complete list of DMS data is available in Supplementary Table 4. We also evaluate against clinical antibody escape susceptibility data from https://covdb.stanford.edu/search-drdb/?form_only. We use the following PDB identifiers: 6VXX, 6VYB, 7CAB, 7BNN, 1RVX, 5FYL, 7TFO, 7PUY, 5EVM, 7TY0, 7TXZ (Supplemental Table 1). Prior models of antibody escape are available from: https://github.com/3BioCompBio/SpikeProSARS-CoV-2 and https://github.com/brianhie/viral-mutation. Multiple sequence alignments are constructed with sequences from: https://www.uniprot.org/uniref/?facets=identity%3A1.0&query=%2A. All source data are provided with this paper.

Human research participants

Policy information about studies involving human research participants and Sex and Gender in Research.

Reporting on sex and gender	N/A
Population characteristics	N/A
Recruitment	N/A
Ethics oversight	N/A

Note that full information on the approval of the study protocol must also be provided in the manuscript.

Field-specific reporting

Please select the one below that is the best fit for your research. If you are not sure, read the appropriate sections before making your selection.

☒ Life sciences ☐ Behavioural & social sciences ☐ Ecological, evolutionary & environmental sciences

For a reference copy of the document with all sections, see nature.com/documents/nr-reporting-summary-flat.pdf

Life sciences study design

All studies must disclose on these points even when the disclosure is negative.

Sample size	All experimental data were used as published in their respective works (using the entirety of samples - where for DMSs every mutation is measured for every position). The viral proteins selected for this study were chosen based on the availability of comprehensive Deep Mutational Scanning data. All Spike data in GISAID is used.
Data exclusions	No data was excluded from this analysis.
Replication	All primary data used in this analysis was obtained from public repositories, and no experimental replication was performed.
Randomization	All experimental data comes from published sources. Randomization is not relevant to the computational analysis of this study, since the model is fully unsupervised and therefore none of the evaluation data was used in training the model.
Blinding	The same model building process was applied to all proteins in this study and required no human interpretation and so no blinding was necessary.

Reporting for specific materials, systems and methods

We require information from authors about some types of materials, experimental systems and methods used in many studies. Here, indicate whether each material, system or method listed is relevant to your study. If you are not sure if a list item applies to your research, read the appropriate section before selecting a response.

Materials & experimental systems

n/a	Involved in the study	
☒	☐	Antibodies
☒	☐	Eukaryotic cell lines
☒	☐	Palaeontology and archaeology
☒	☐	Animals and other organisms
☒	☐	Clinical data
☒	☐	Dual use research of concern

Methods

n/a	Involved in the study	
☒	☐	ChIP-seq
☒	☐	Flow cytometry
☒	☐	MRI-based neuroimaging
