# Supplementary material for: Learning from prepandemic data to forecast viral escape
Source: Nature. 2023 Oct 11;622(7984):818–25. doi: 10.1038/s41586-023-06617-0 (PMC10599991; doi:10.1038/s41586-023-06617-0)
Supplement: Supplementary file 12 — Acknowledgements for all GISAID sequences. [file 41586_2023_6617_MOESM12_ESM.pdf]

SUPPLEMENTAL TABLE

Data Availability

GISAID Identifier:	EPI_SET_230814cp
doi:	10.55876/gis8.230814cp

All genome sequences and associated metadata in this dataset are published in GISAID's EpiCoV database. To view the contributors of each individual sequence with details such as accession number, Virus name, Collection date, Originating Lab and Submitting Lab and the list of Authors, visit 10.55876/gis8.230814cp

Data Snapshot

- EPI_SET_230814cp is composed of 15,667,960 individual genome sequences.
- The collection dates range from 2010-12-06 to 2023-06-06;
- Data were collected in 221 countries and territories;
- All sequences in this dataset are compared relative to hCoV-19/Wuhan/WIV04/2019 (WIV04), the official reference sequence employed by GISAID (EPI_ISL_402124). Learn more at https://gisaid.org/WIV04.
